# Bread Wheat With High Salinity and Sodicity Tolerance

**DOI:** 10.3389/fpls.2019.01280

**Published:** 2019-10-22

**Authors:** Yusuf Genc, Julian Taylor, Graham Lyons, Yongle Li, Judy Cheong, Marie Appelbee, Klaus Oldach, Tim Sutton

**Affiliations:** ^1^South Australian Research and Development Institute, Adelaide, SA, Australia; ^2^School of Agriculture, Food and Wine, University of Adelaide, Adelaide, SA, Australia; ^3^LongReach Plant Breeders, Adelaide, SA, Australia

**Keywords:** chloride, ionic, osmotic, salinity, sodicity, sodium, sodium humate, tolerance

## Abstract

Soil salinity and sodicity are major constraints to global cereal production, but breeding for tolerance has been slow. Narrow gene pools, over-emphasis on the sodium (Na^+^) exclusion mechanism, little attention to osmotic stress/tissue tolerance mechanism(s) in which accumulation of inorganic ions such as Na^+^ is implicated, and lack of a suitable screening method have impaired progress. The aims of this study were to discover novel genes for Na^+^ accumulation using genome-wide association studies, compare growth responses to salinity and sodicity in low-Na^+^ bread Westonia with *Nax1* and *Nax2* genes and high-Na^+^ bread wheat Baart-46, and evaluate growth responses to salinity and sodicity in bread wheats with varying leaf Na^+^ concentrations. The novel high-Na^+^ bread wheat germplasm, MW#293, had higher grain yield under salinity and sodicity, in absolute and relative terms, than the other bread wheat entries tested. Genes associated with high Na^+^ accumulation in bread wheat were identified, which may be involved in tissue tolerance/osmotic adjustment. As most modern bread wheats are efficient at excluding Na^+^, further reduction in plant Na^+^ is unlikely to provide agronomic benefit. The salinity and sodicity tolerant germplasm MW#293 provides an opportunity for the development of future salinity/sodicity tolerant bread wheat.

## Highlight

A new bread wheat germplasm accumulates high levels of sodium, and yet is more tolerant to salinity and sodicity than the bread wheats with better sodium exclusion ability.

## Introduction

Soil salinity and sodicity severely constrain crop production in Australia and worldwide. The total global area of saline and sodic soils is estimated to be around 830 million hectares, more than 6% of the world’s land (Martinez-Beltran and Manzur, 2005) and rising (Acosta-Motos et al., 2017; Bennett et al., 2013; Shrivastava and Kumar, 2015). Indeed, it is estimated that over 50% of global arable land will be salinized by 2050 (Jamil et al., 2011). Although the actual cost from lost agricultural production is hard to quantify, and varies with crop species, timing, duration, and severity of the stress, it is apparent that losses in yield and profit are significant (McDonald et al., 2012). Yield reductions of 50% in durum wheat under dryland salinity (James et al., 2012), 88% in bread wheat under high irrigation salinity (Jafari-Shabestari et al., 1995), and 70% under sodicity have been reported (Rengasamy, 2002). These studies highlight the scale of lost productivity on saline and sodic soils, and the great opportunity if yield in these environments can be improved.

When cropping on saline and sodic soils, there are limited options to raise productivity, and they are complementary (Singh et al., 2016): (i) soil management and (ii) plant breeding. Despite the potential of the plant breeding approach (Grewal et al., 2004; Genc et al., 2007; Munns et al., 2012), progress in breeding cereal cultivars with salinity or sodicity tolerance has been slow (Noble and Rogers, 1992; Shahbaz and Ashraf, 2013; Iqbal et al., 2014; Volkov and Beilby, 2017). This is often attributed to the genetic and physiological complexities of the salt tolerance trait, and lack of a reliable and rapid screening assay (Flowers, 2004; Colmer et al., 2005; Kumar and Mosa, 2015; Genc et al., 2016; Almeida et al., 2017). Moreover, elite germplasm may not include genes able to confer worthwhile salt/sodicity tolerance, and introgression from wild wheat relatives and/or genetic engineering may be required for step change progress to be achieved (Glenn et al., 1997; Wang et al., 2003; Colmer et al., 2005; Colmer et al., 2006; Wang et al., 2007; Shavrukov et al., 2009; Munns et al., 2012; Deinlein et al., 2014; Zhang et al., 2016).

An example of the use of a wild relative is the work of Richard James and his colleagues (James et al., 2012; Munns et al., 2012), who introgressed Na^+^ exclusion genes *Nax1* and *Nax2* from the diploid bread wheat ancestor *Triticum monococcum* L. (C68-101) into durum wheat Tamaroi. *Nax1* removes Na^+^ from the xylem in roots and leaf sheaths, while *Nax2* removes Na^+^ from xylem in the roots only (James et al., 2011). Tamaroi with *Nax2* showed lower leaf Na^+^ concentration and achieved higher grain yield under salinity (Munns et al., 2012) and sodicity (Genc et al., 2016). These two genes were also transferred from durum wheat into bread wheat cv. Westonia, and subsequently shown to reduce leaf 3 Na^+^ concentration (James et al., 2011). Recent saline field trials with three Westonia-Nax2 and two Westonia-Nax1 lines indicated, compared to Westonia, 11% yield increase in 2009 but 6.5% decrease in 2011 in one of the Westonia-Nax2 lines (Westonia- Nax2-5924) (**Tables S1** and **S3**; Setter et al., 2016). These results are not conclusive. Therefore, there is a need to verify the effects of these genes in bread wheat in controlled environment studies involving salinity and sodicity, especially as bread wheat has much greater Na^+^ exclusion than durum wheat (Genc et al., 2016). Despite their potential for improving salinity tolerance (reviewed in Colmer et al., 2006), wild relatives and landraces of bread wheat largely remain an untapped resource. In the early 2000s salinity tolerant bread wheat germplasm lines W4909 and W4910, derived from wild relatives, were developed by Richard Wang and his colleagues (Wang et al., 2003). However, these germplasm lines have not been exploited in breeding programs.

Sodicity, of which high Na^+^ is the key component, affects greater land area than salinity (Rengasamy and Olsson, 1991; Rengasamy, 2002; Rengasamy, 2006), but there has been little specific research on sodicity and mechanisms of tolerance (Pearson and Bernstein, 1958; Sharma, 1986, Sharma, 1991; Rajpar et al., 2004; Saqib et al., 2007). This is unsurprising as screening for sodicity tolerance has been difficult in laboratory or glasshouse environments (Singh et al., 2002), which are needed to test large numbers of accessions in a relatively controlled manner. Problems with current screening methods include (i) very high pH of sodic soils, hence difficulty of separating pH effects from those of Na^+^ toxicity, (ii) inability to control soil composition when sourced from field sites, and (iii) months of waiting before pH stabilizes, and thereafter the possibility of toxicity from excess salt (sodium bicarbonate) not adsorbed at cation exchange sites (Singh et al., 2002). A recently developed soil-based screening method, using Na^+^-humate as a surrogate for sodicity (Genc et al., 2016), avoids these issues and enables screening of a large number of accessions. We utilised this method in order to determine genotypic variation in Na^+^ exclusion in commercial bread wheat varieties and assess its importance to sodicity and salinity tolerance.

In efforts to develop selection tools that can be applied in breeding, genetic markers for the Na^+^ exclusion trait in cereals have been identified by QTL mapping of bi-parental populations (Genc et al., 2010; Shavrukov et al., 2011; Masoudi et al., 2015; Hussain et al., 2017). However, most of these genetic markers have not been implemented in breeding programs as they mostly represent small effect loci that require extensive validation in alternative genetic backgrounds under appropriate conditions. An alternative to aforementioned QTL mapping is association mapping or genome-wide association studies (GWAS). Two major advantages of association mapping over QTL mapping are (i) a much larger and more representative gene pool can be surveyed, and (ii) it bypasses the time-consuming and expensive process of constructing bi-parental mapping populations (Neumann et al., 2011). Although GWAS can be applied to a variety of plant species and conditions, only a few studies have reported on Na^+^ exclusion and/or salt tolerance in rice (Kumar et al., 2015; Patishtan et al., 2017; Shi et al., 2017), wheat (Turki et al., 2015; Oyiga et al., 2018), and barley (Long et al., 2013; Fan et al., 2016). Given these limited studies, there is clearly a role of GWAS to detect novel genes/alleles associated with salinity/sodicity tolerance, which can be deployed in breeding programs.

The aims of this study were to (i) identify novel genes/alleles for Na^+^ accumulation which may be involved in osmotic stress/tissue tolerance, (ii) compare growth responses to salinity and sodicity in low-Na^+^ bread wheat Westonia with *Nax1* and *Nax2* genes and high-Na^+^ bread wheat Baart-46, and (iii) evaluate growth responses to salinity and sodicity in bread wheats with varying leaf Na^+^ concentrations.

## Materials and Methods

### Plant Material

Experiment 1 included 100 bread wheat entries (**Table S1**) forming a diversity panel based on differential growth, yield, and Na^+^ exclusion (Richards et al., 1987; Shavrukov et al., 2006; Genc et al., 2007; James et al., 2011; Setter et al., 2016). Most entries were released Australian varieties dating back to 1901 and the remainder were historical varieties from Egypt, Germany, India, Mexico, and USA. Twelve durum wheat entries (*Triticum turgidum* subsp *durum*) were also included as checks. As durum wheats generally accumulate higher Na^+^ levels than bread wheats, we included two unique durum lines with low Na^+^ concentration (cv. Tamaroi with the *Nax2* and breeding line WID902 with both *Nax1* and *Nax2*). In Experiment 2, there were four bread wheat (*Triticum aestivum* L.) entries (Westonia, Westonia with Na^+^ exclusion gene *Nax1* (Westonia-Nax1-5907), Westonia with Na^+^ exclusion gene *Nax2* (Westonia-Nax2-5924), and Baart-46). The *Nax1* (*TmHKT1;4-A2*) and *Nax2* (*TmHKT1;5-A*) genes have been previously characterized (James et al., 2006; Byrt et al., 2007). As bread wheat cv. Westonia represents low Na^+^ wheat genotypes, a bread wheat genotype representing high-Na^+^ wheat genotypes, cv. Baart-46 (Genc et al., 2007), was also included for comparison. In experiment 3, there were 20 bread wheat entries (representing the range in leaf Na^+^ concentration in Experiment 1), four durum wheat entries, and a barley entry Clipper as checks. In Experiment 4, wheat lines cv. Mace, and doubled-haploid lines from a Mace/W4909 cross with low leaf Na^+^ concentration (MW#28 and MW#491), and high leaf Na^+^ concentration (MW#293 and MW#451) were used.

### Growth Medium, Treatments, Seedling Establishment, and Growth Conditions

All four experiments used University of California potting mix, described previously (Genc et al., 2016), with biological replicates varying from four to nine. In Experiment 1, as plants were grown until heading to determine Na^+^ concentration in leaves at a single level of sodicity (8 g kg^-1^ Na^+^-humate), four plants per pot were grown in 3 kg capacity pots to enable testing of more wheat entries. There were four replications. In Experiment 2, there were five salinity (0, 50, 100, 150, and 200 mM NaCl) and four sodicity (2, 4, 8, and 16 g kg^-1^ Na^+^-humate) levels which were replicated four times. As plants were grown to maturity, 4 kg capacity pots were used as described in Genc et al. (2016). There were three plants per pot. In Experiment 3, as plants were grown to maturity, 4 kg capacity pots were used. There were four replications and three plants per pot grown under control, sodicity (8 g kg^-1^ Na^+^-humate), and salinity (100 mM NaCl). In Experiment 4, plants were grown to heading or maturity under control and salinity in 4 kg capacity pots. There were five (nutrient analysis) or nine (gene expression) replications, and three plants per pot. At heading, penultimate leaves were sampled for gene expression, while in the other set penultimate leaves were sampled for elemental analysis and plants were grown to maturity.

### Plant Traits Measured

As described earlier (Genc et al., 2016), in all experiments the pots were weighed daily, and watered to field capacity (7.4%w/w) until heading and 10% thereafter with milli-Q water. Weekly incremental water uptake was used to estimate growth rates (a measure of osmotic stress tolerance; Munns and Tester, 2008) in Experiments 1 and 3 (Genc et al., 2016). At heading (main culm fully emerged), penultimate leaves were sampled for analysis of Na^+^, potassium (K^+^), calcium (Ca^2+^), magnesium (Mg^2+^), and Cl^-^ (Genc et al., 2016). Handling of leaf samples and analytical methods used in nutrient analyses were described earlier (Genc et al., 2016). At maturity, grain yield per plant was determined. For across and within species comparisons, relative grain yield (the ratio of yield at an individual stress level to that under nil stress and expressed as percent) was also calculated.

### Candidate Gene Selection, Primer Design, and Gene Expression in Penultimate Leaves Under Control and Salinity

Candidate genes and previously reported genes were selected based on published literature and findings of the present study (**Table S2**). Primers for qPCR were sourced from previous studies or designed against relevant cDNA sequences from NCBI (**Table S2**). Specific qPCR amplification was confirmed by obtaining a single distinct peak in melt curve analysis, and qPCR product sequencing.

Total RNA was isolated from penultimate leaves using Spectrum Plant Total RNA kit (Sigma) with an on-column DNase treatment. SuperScript III Reverse Transcriptase kit (Life Technologies) was used to synthesize the cDNA. The reaction contained 500 ng purified RNA from each sample in a final reaction volume of 20 µl, performed according to manufacturer’s instructions. The qPCR assays were prepared according to manufacturer’s instructions using PrecisionFAST qPCR mix (Primer Design Ltd). Amplification were performed in a QuantStudio 6 Flex Real-Time PCR System (Thermo Fisher) with 3 min of 95°C followed by 40 cycles of 3s at 95°C, 20s at 60°C, and fluorescent acquisition at 60°C, followed by melt curve analysis. Three wheat genes, encoding actin, glyceraldehyde 3-phosphate dehydrogenase (GAPDH), and elongation factor 1-alpha (EF1a) were used together for normalization of target gene expression. Purified PCR products of target genes, covering six orders of magnitude, were used to construct a standard curve in relation to the cycle threshold (Ct) value from which the actual copy number per microgram of RNA was obtained.

### Experimental Design and Statistical Analysis

In Experiments 1 and 3 the number of pots required exceeded the size of a single growth room and consequently two growth rooms with identical settings were used (four replication in each experiment). Variety by treatment combinations were allocated to pots within each growth room using a randomized complete block design (RCBD) with two replicates. For Experiments 2 and 4, the variety by treatment combinations were allocated to pots in a single growth room using an RCBD with four replicates of each combination. To overcome the problem of variance heterogeneity, all leaf Na^+^ and some Cl^-^ data were log-transformed prior to model fitting. For each experiment, analysis of measured elemental and grain yield related traits was conducted using linear mixed models that appropriately captured sources of treatment and variety variation as well as environmental variation associated with the experiments. In each model the fixed component contained term accounting for variety and treatment main effects as well as variety by treatment interaction effects. To appropriately account for extraneous variation, physical design constraints such as multiple growth rooms and replicates within growth rooms was accounted for using random effects. For any given trait, perceived observational outliers were down-weighted using a simple indicator covariate random effect term (Gumedze et al., 2010). From each of the fitted models best linear unbiased estimates (BLUEs) and standard errors for the variety by treatment interaction means were extracted for summary. For traits analysed from Experiment 1 the Honest Significant Difference (HSD) at *P =* 0.05 was used to control the familywise error rate when comparing between means. For traits analysed from Experiment 2, 3, and 4 where there was a reduced number of variety by treatment combinations, a Least Significant Difference (LSD) at *P =* 0.05 was calculated and used to compare variety by treatment means.

Similar to Genc et al. (2016), plant water uptake in Experiment 1 and 3 was statistically assessed using weekly incremental water use from 15 days of transplanting to heading of each variety. To determine differences in the rate of water use between the levels of salinity or sodicity treatments across varieties, a longitudinal regression analysis was conducted using a linear mixed model. In this model, the fixed component contained terms to model the intercept and linear slope of the water use over time for each of the variety by treatment combinations. Additional non-linearity was modelled using a random cubic smoothing spline term (Verbyla et al., 1999). For each model the estimated variety by treatment linear coefficients were extracted and an LSD at *P =* 0.05 was calculated to provide a comparison between estimates. Model based prediction curves of incremental water use were also calculated for graphical summary.

All linear mixed modelling of grain yield, elemental, and water use traits was computationally conducted using the flexible ASReml-R software (Butler et al., 2009) available as a package in the R statistical computing environment (R Core Team, 2018).

### Genome-Wide Association Study and Identification of Candidate Genes

DNA of 100 bread wheat entries was extracted from leaf tissue using the phenol/chloroform extraction method (Williams et al., 2002) and genotyped with the 90K wheat SNP array (Wang et al., 2014). Population structure was estimated using ADMIXTURE (v1.23) software (Alexander and Lange, 2011) which uses a model-based algorithm to estimate the ancestry of individuals. Cross-validation was used to determine the most likely number of clusters to be used in the subsequent modelling. For the 100 bread wheat entries, the BLUEs of Na^+^ extracted from the fitted model of Experiment 1 were used in genome-wide association mapping, based on 41,035 SNP markers with minor allele frequency (MAF)>0.05 and <50% missing call rate. For each SNP, a mixed-linear model (MLM) was fitted where the fixed component of the model contained a numerical version of the SNP as well as a covariate to adjust for the confounding effects of population structure. The MLM also contained a random effect for the lines with an assumed variance structure equivalent to the kinship matrix centred using the IBS method and then compressed to optimum groups. This then allowed the P3D (population parameters previously determined) compressed MLM method to be used to speed up computation time (Zhang et al., 2010). From each of the fitted models, SNP effects were assessed using a significant p-value threshold set at *P =* 8.91e-5 equivalent to α level of 0.05 after adjusted Bonferroni correction using the simple*M* method (Gao et al., 2008). Bonferroni correction assumes that the hypothesis tests are independent which is not true due to linkage disequilibrium among the SNP in GWAS study. Briefly, the simple*M* method calculated the effective number of independent test using principal component analysis based on the SNP data. Subsequently, the number of test in the Bonferroni correction formula was replaced by the effective number of independent test. All genome wide association mapping and assessment was computationally conducted using TASSEL software (Bradbury et al., 2007). Based on the physical position of the markers and high confidence gene content in the Chinese Spring Reference Genome (IWGSC RefSeq v1.0), genes located within a 700 kb region flanking the significant SNP were reported. Genes were annotated using IWGSC RefSeq v1.0 annotation available in URGI (https://wheat-urgi.versailles.inra.fr/Seq-Repository/Annotations).

## Results

### Experiment 1. Genome-Wide Association Mapping of Na^+^ Accumulation in 100 Bread Wheat Entries

**Figure 1:**
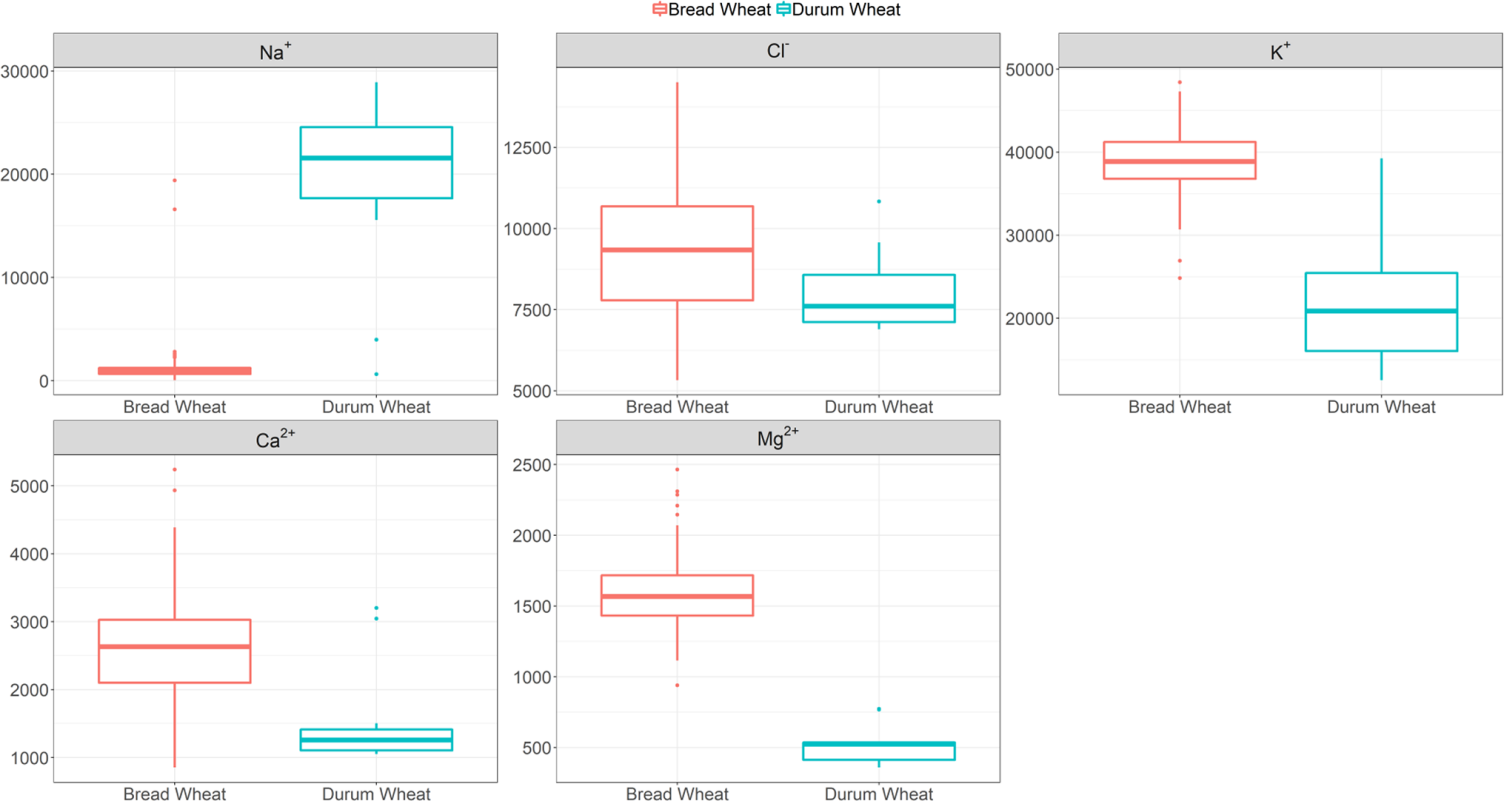
Boxplots of leaf Na^+^ (back-transformed), Cl^-^, K^+^, Ca^2+^ and Mg^2+^ concentrations (mg kg^-1^ DW) at heading in 100 bread wheat entries, 12 durum wheat entries and a barley cultivar grown under sodicity (8 g kg^-1^ Na^+^-humate) in Experiment 1 (n = 4). See **Table S3** for individual responses. The box represents the middle 50% of the distribution (the median is drawn as the solid line within the box) with whiskers extending to the lowest/highest value within 1.5* IQR (Inter-Quartile Range). Values outside this range are plotted separately.

Given the benefits of Na^+^ exclusion under sodicity but not salinity observed in our initial study (Genc et al., 2016), here we screened a bread wheat diversity set under sodicity to determine genetic variation for Na^+^ exclusion. **Figure 1** and **Table S3** show that there is genetic variation in Na^+^ exclusion (*P* < 0.001), but almost all elite bread wheat entries had high Na^+^ exclusion (approx. <2,000 mg Na^+^ kg^-1^ DW), compared to typical durum wheat entries (approx. 15,000–30,000 mg Na^+^ kg^-1^ DW). Leaf Na^+^ concentrations in bread wheats varied from 50 mg kg^-1^ DW in Westonia-*Nax2* to 2,800 mg kg^-1^ DW in cv. Olympic. The only exceptions to this were two bread wheat germplasm lines (MW#451 and MW#293; approx. >15,000 mg Na^+^ kg^-1^ DW) which grouped with the durum wheats. The presence of Na^+^ exclusion genes *Nax1* and *Nax2* in durum wheat (*Nax1* and *Nax2* in WID902; *Nax2* in Tamaroi) was associated with much lower Na^+^ concentrations (approx. 600–4,000 mg kg^-1^ DW) than in durum wheats lacking these genes (**Figure 1**, **Table S3**).

Calcium, K^+^, and Mg^2+^ concentrations varied significantly amongst the bread wheat entries (850–5,240, 24,820–48,400, and 940–2,460 mg kg^-1^ DW, respectively), but variations were much lower than those observed for Na^+^ concentration, and values were lower in durum wheats (**Figure 1**, **Table S3**).

### Single Nucleotide Polymorphism Markers Significantly Associated With Na^+^ Concentration in 100 Bread Wheat Entries

**Figure 2:**
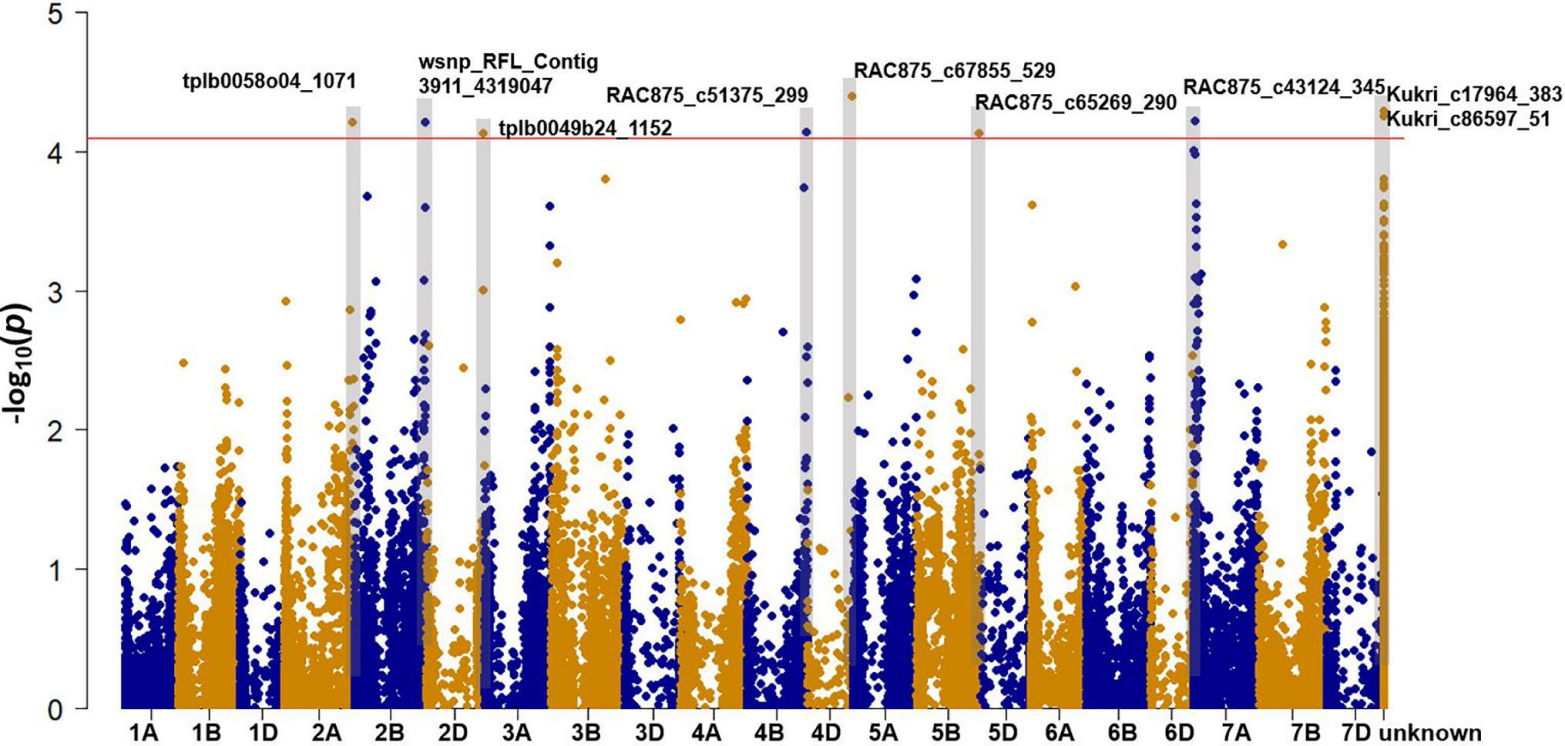
Manhattan plot showing the association signals for leaf Na^+^ concentration using 100 bread wheat entries grown under sodicity (8 g kg^-1^ Na^+^-humate) in Experiment 1 (n = 4). The x-axis indicates the physical location of SNP markers along each wheat chromosome; the y-axis shows the *P*-value of SNP markers for the association test on a log scale. The horizontal red line indicates the significance threshold (P-value = 8.91e-5). The marker names of SNPs above the threshold are shown.

Genome-wide association mapping was performed with 41,035 SNP markers (MAF > 5%) in 100 bread wheat entries taking population structure effect (K = 3) into account (**Figure S1**). We identified nine SNPs significantly (*P*-value < 8.91e-5) associated with leaf Na^+^ concentration (log-transformed) (**Table S4**, **Figure 2**). Using the IWGSC RefSeq v1.0, seven SNPs were mapped to chromosomes 2A, 2B, 2D, 4B, 4D, 5B, and 7A We examined the high confidence (HC) genes located within 700Kb left and right of each significant SNP, and identified four candidate genes with potential functions in Na^+^ accumulation/exclusion. These were calcium-transporting ATPase (TraesCS4D01G343200.1), Na^(+)^/H^(+)^ antiporter NhaB (TraesCS4D01G344200.1), Aquaporin*TIF1-4* (TraesCS4D01G344300.1), and Aquaporin *PIP2* (TraesCS4B01G362300.1). It is notable that MW#451 and MW#293 contain the rare alleles that gave rise to extremely high Na^+^ concentrations (allele effects for SNPs shown in **Table S5**). We did not identify any mapped significant SNPs in proximity to the Na^+^ exclusion genes *Nax1 (2A)* and *Kna1* (4D; *TaHKT1;5-D*) which is homologous to *Nax2* in the diploid bread wheat ancestor *T. monococcum*.

### Experiment 2. Growth Responses of Westonia, Westonia-*Nax1*, Westonia-*Nax2*, and Baart-46 Under a Range of Salinity and Sodicity

Given the lack of SNPs associated with Nax genes observed in Experiment 1, we proceeded with a phenotype experiment to investigate further.

A regression analysis of weekly incremental water use indicated that all wheat lines had reduced water uptake and consequently reduced growth rates under increasing salinity and sodicity (**Figure S2**, **Table S6**), and reductions were greater under salinity than sodicity. Under salinity, there were no differences amongst the Westonia lines, while Baart-46 had higher growth rates than the Westonia lines until 100 mM NaCl was reached. Under sodicity, Westonia-*Nax1* had higher growth rate than Westonia-*Nax2* at 8 g kg^-1^ soil Na^+^-humate, but there were no other significant differences amongst Westonia lines at other levels. Baart-46 had higher growth rates than Westonia lines at all levels of sodicity (**Table S6**).

**Figure 3:**
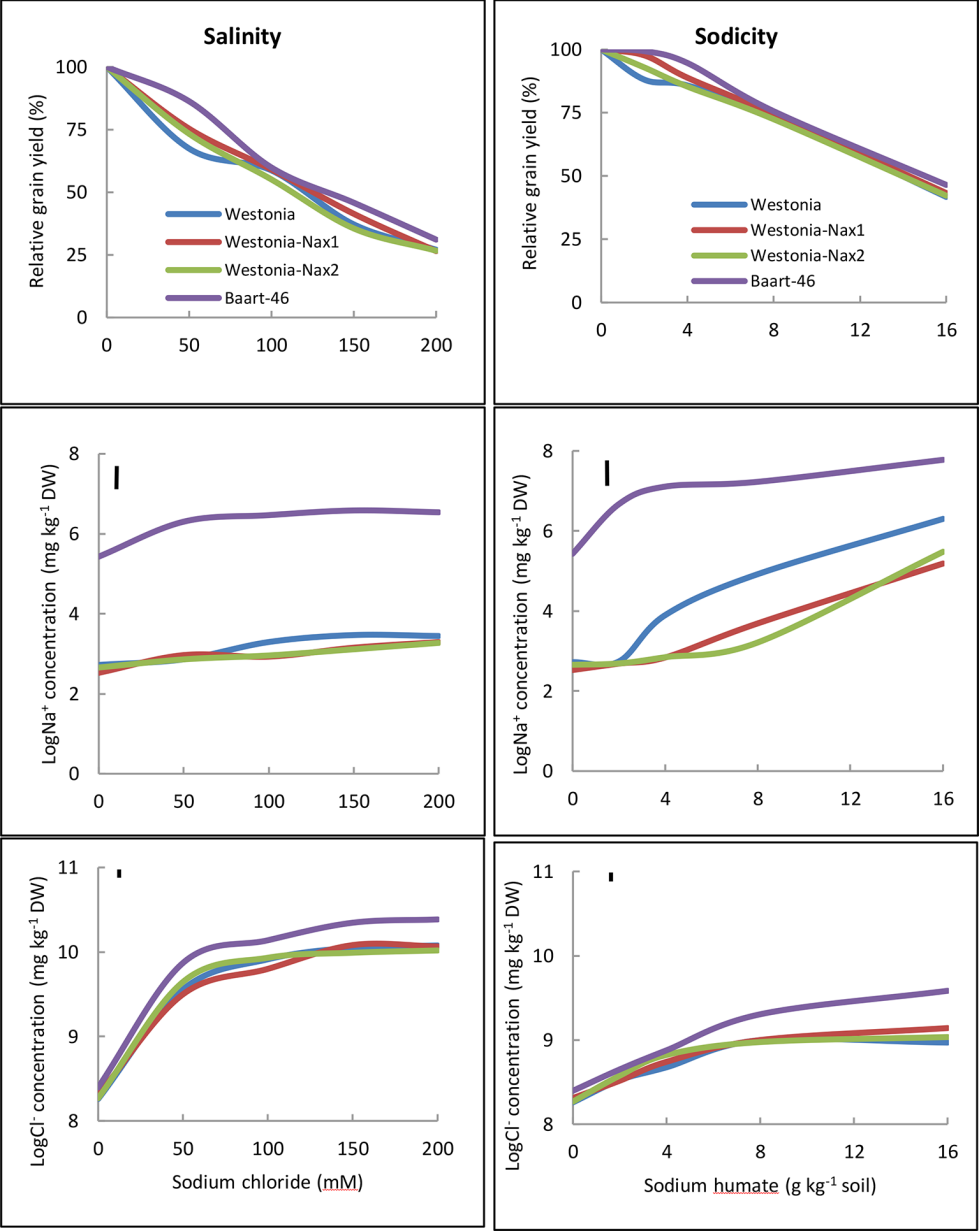
Relative grain yield (%) (salinity or sodicity tolerance), and best linear unbiased estimates for leaf Na^+^ and Cl^-^ concentrations in wheat cv. Westonia, Westonia-*Nax1*, Westonia-*Nax2* and Baart-46 under different levels of salinity (left panels) and sodicity applied as Na^+^ humate (right panels) in Experiment 2 (n = 4). The vertical bars indicate Least Significant Difference test value at *P =* 0.05 for variety x treatment interaction. See **Table S8** for back-transformed Na^+^ and Cl^-^concentrations for comparisons with published data.

Penultimate leaf Na^+^ concentrations were higher under sodicity than salinity (**Figure 3**, **Tables S7** and **S8**). Baart-46 maintained higher Na^+^ concentrations than Westonia and Nax lines under both stresses (**Figure 3**, **Tables S7** and **S8**). As compared to Westonia, the presence of *Nax1* and *Nax2* genes was associated with reduced Na^+^ concentrations (**Figure 3**, **Tables S7** and **S8**). Reductions were similar for both genes, and became more pronounced at higher rates of salinity and sodicity, reaching maxima of 72–82% and 32–34% reductions at 8 g kg^-1^ Na^+^-humate and 100 mM NaCl, respectively. Chloride concentrations were much higher under salinity than sodicity, and higher in Baart-46 than Westonia and Nax lines, the latter group being similar to each (**Figure 3**, **Tables S7** and **S8**). For other cations, Ca^2+^ concentrations were lower under sodicity than salinity, Mg^2+^concentrations were similarly low under both salinity and sodicity, while K^+^ concentrations remained unaffected, either by salinity or sodicity (**Table S7**). The most notable genetic differences were higher K^+^, and lower Ca^2+^ and Mg^2+^ in Baart-46 than the other three lines under salinity and sodicity. Reduced Na^+^ concentrations in the Westonia Nax lines was not accompanied by higher grain yield, with small grain yield increases observed only under moderate salinity and low sodicity (6–9% at 50 mM NaCl and 5–10% at 2 g kg^-1^ Na^+^-humate) (**Table 1**). At these salinity and sodicity rates, despite much higher Na^+^ concentrations than Westonia and Nax lines, cv. Baart-46 was similar or higher for grain yield (**Table 1**; **Figure 3**). There were no other notable differences in salinity or sodicity tolerance (relative grain yield %) amongst the four bread wheat lines and salinity/sodicity rates (**Table 1**).

**Table 1:** Best linear unbiased estimates for grain yield in bread wheat *Triticum aestivum* L.) cv. Westonia, Westonia-*Nax1*, Westonia-*Nax2*, and Baart-46 under different levels of salinity and sodicity in Experiment 2 (n = 4).

Salinity (mM NaCl)	Grain yield plant^-1^(g)			
	Westonia	West-*Nax1*	West-*Nax2*	Baart-46
0	5.450	5.375	5.625	7.475
50	3.675	4.050	4.125	6.450
100	3.200	3.175	3.100	4.475
150	2.025	2.225	2.000	3.425
200	1.475	1.425	1.500	2.325
LSD* Variety x Treat		0.554		
Sodicity (g kg^-1^ soil Na^+^ humate)	Westonia	West-*Nax1*	West-*Nax2*	Baart-46
0	5.450	5.375	5.625	7.475
2.0	4.800	5.250	5.225	7.425
4.0	4.675	4.775	4.800	7.075
8.0	4.025	4.000	4.075	5.650
16.0	2.275	2.325	2.375	3.475
LSD* Variety x Treat		0.532		

*LSD refers to least significant difference at P = 0.05 for species x treatment interaction.

### Experiment 3. Effects of a Wide Range of Na^+^ Exclusion on Salinity and Sodicity Tolerance in 20 Bread Wheats, Three Durum Wheats, and a Barley Entry

To expand our investigation in Experiment 2, we studied a wider range of germplasm.

Regression analyses of incremental water use up to heading stage indicated that growth rates were reduced significantly by salinity and sodicity in all bread wheats, durum wheats, and barley cv. Clipper, with a much lower reduction occurring in bread wheat germplasm line MW#293 which had the highest growth rate under salinity and sodicity (**Table S9**, **Figure S3**). The *Nax2* gene in durum wheat Tamaroi was associated with a non-significant increase in growth rate under sodicity, while there was no benefit under salinity. Barley cv. Clipper had generally higher growth rates than the averages of bread wheats under salinity and sodicity. There were also differences in growth rates under control.

**Figure 4:**
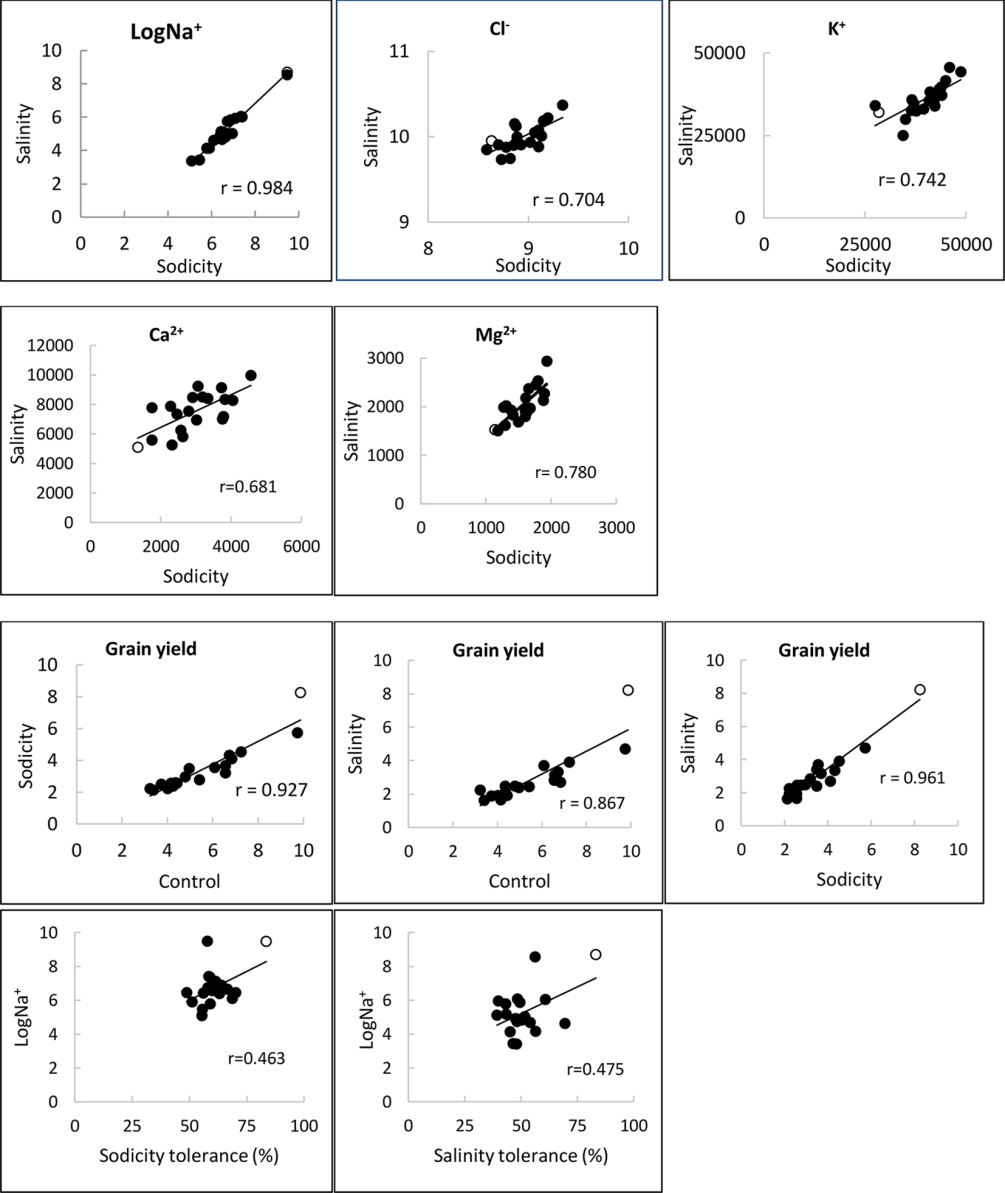
Relationships of Na^+^, Cl^-^, K^+^, Ca^2+^ and Ma^2+^ concentrations (mg kg^-1^ DW), and grain yield (g plant^-1^) under control, salinity (100 mM NaCl) and sodicity (8 g kg^-1^ Na^+^-humate) in 20 bread wheat entries in Experiment 3 (n = 4) (r = 0.561,*df =* 18, *P* < 0.01). MW#293 is shown as an empty circle.

Salinity and sodicity increased leaf Na^+^ concentrations in all entries, and concentrations were higher under sodicity than salinity (**Table 2**). Amongst the commercial wheats, older cultivars such as Federation and Baart-46 had higher Na^+^ concentrations (approx. 430–460 and 1,700-1,800 mg kg^-1^ DW under salinity and sodicity) than modern cultivars (approx.< 400 and 1,200 mg kg^-1^ DW under salinity and sodicity) (**Table 2**). However, none of the cultivars had Na^+^ concentrations as high as the two novel germplasm lines (MW#451 and MW#293) derived from wild relatives of bread wheat (*Thinopyrum junceum and Aegilops speltoides*) (back-transformed averages of these two lines; approx. 5,600 and 13,000 mg Na^+^ kg^-1^ DW under salinity and sodicity, respectively) (**Table 2**). There was a significant correlation between Na^+^ concentration under salinity and sodicity (r = 0.984, *df =* 18, *P* < 0.01) (**Figure 4**). Barley entry Clipper had Na^+^ concentrations (approx. 7,000 and 17,000 mg kg^-1^ DW under salinity and sodicity) as high as those in high-Na^+^ wheat germplasm lines MW#293 and MW#451, while durum wheats Yawa and Tamaroi had overall the highest Na^+^ concentrations (approx. 6,700 and 11,400 under salinity; 20,600 and 32,700 mg kg^-1^ DW under sodicity, respectively). As expected, concentrations of Cl^-^ rose significantly under salinity in all entries, and the increases were similar for the three species (**Table 2**).

**Table 2:** Best linear unbiased estimates for leaf Na^+^and Cl^-^ concentrations (mg kg^-1^ DW) in 20 bread wheat entries (*Triticum aestivum* L.), three durum wheat entries (*Triticum turgidum* subsp *durum* cv. Tamaroi, Tamaroi-*Nax2*, and Yawa), and one barley cultivar (*Hordeum vulgare* L.cv. Clipper) under control, salinity (100 mM NaCl), and sodicity (8 g kg^-1^ Na^+^-humate) in Experiment 3 (n = 4).

Bread wheat	*LogNa^+^			*LogCl^–^			**Back-transformed Na^+^			**Back-transformed Cl^–^		
	control	sodicity	salinity	control	sodicity	salinity	control	sodicity	salinity	control	sodicity	salinity
Longreach Cobra	3.0	5.1	3.4	8.6	9.0	9.9	21	162	30	5682	8250	20680
Westonia	2.8	5.4	3.4	8.5	8.8	9.7	16	232	31	4805	6744	17077
Krichauff	2.8	5.8	4.2	8.6	8.7	9.9	17	325	64	5303	6007	20118
Mace	3.2	5.9	4.1	8.7	9.1	9.9	24	365	63	6149	8972	19653
Wyalkatchem	3.2	6.1	4.6	8.7	9.1	10.1	24	443	100	6143	8619	23198
Axe	3.8	6.4	4.9	8.7	8.8	9.9	46	601	133	5762	6499	19540
Halberd	2.7	6.4	4.8	8.8	8.9	10.1	16	606	126	6810	7158	24986
Beckom	3.0	6.4	4.7	9.1	9.2	10.2	20	620	114	8891	9852	27560
Yitpi	3.1	6.4	5.2	8.4	8.6	9.9	23	622	174	4588	5340	18983
Condor	2.6	6.5	4.7	8.9	9.2	10.2	13	658	106	7611	9428	26562
Kharchia-65	4.0	6.6	5.1	8.8	8.9	10.2	56	705	165	6363	7071	25632
AGT Katana	2.9	6.6	4.8	8.6	8.9	9.9	18	766	125	5325	7514	20052
Drysdale	5.1	6.7	5.8	8.5	8.7	9.7	162	847	318	4864	6177	16864
Pitic-62	3.1	6.9	5.9	9.2	9.3	10.4	22	976	351	9788	11421	31979
Correll	3.7	7.0	5.0	8.9	9.1	10.1	39	1053	151	7425	8953	23822
Hartog	5.6	7.1	5.9	8.9	8.9	9.9	274	1213	383	7043	6993	19902
Federation	5.2	7.4	6.1	8.6	8.9	10.0	178	1571	425	5597	7219	22040
Baart-46	5.6	7.4	6.0	8.8	9.1	10.0	279	1651	417	6534	9231	22194
MW#293	6.8	9.5	8.7	8.6	8.6	10.0	884	12939	6045	5337	5608	20961
MW#451	7.1	9.5	8.6	8.8	8.9	9.9	1216	13063	5187	6424	7192	20813
Tamaroi-*Nax2*	5.8	8.3	7.5	8.4	8.6	10.0	317	4197	1853	4543	5657	21773
Yawa	7.6	9.9	8.7	8.6	8.3	10.2	1922	19339	5949	5435	3896	27656
Tamaroi	7.8	10.3	9.2	8.5	8.6	10.2	2510	29025	10228	5050	5372	26069
Clipper	8.0	9.6	8.7	9.3	9.4	10.0	3099	15128	6042	11089	11797	22533
LSD control vs. sodicity		0.4			0.2							
LSD control vs. salinity			0.4			0.1						

*Na^+^ and Cl^–^ concentration data were transformed to natural logarithms. LSD refers to Least Significant Difference at P = 0.05 for species x treatment interaction. **Back-transformed Na^+^ and Cl^–^ values are also presented to aid comparisons with previously published data.

The other cations measured were K^+^, Ca^2+^, and Mg^2+^. Reduced concentrations of K^+^ and Ca^2+^ under sodicity and salinity were moderate, while Mg^2+^ was significantly reduced, especially under sodicity (**Tables S9** and **S10**). Durum wheats Yawa and Tamaroi and barley entry Clipper exhibited mild Mg^2+^ deficiency symptoms under sodicity. There were significant correlations between salinity and sodicity for concentrations of K^+^, Ca^2+^, and Mg^2+^ (**Figure 4**).

**Figure 5:**
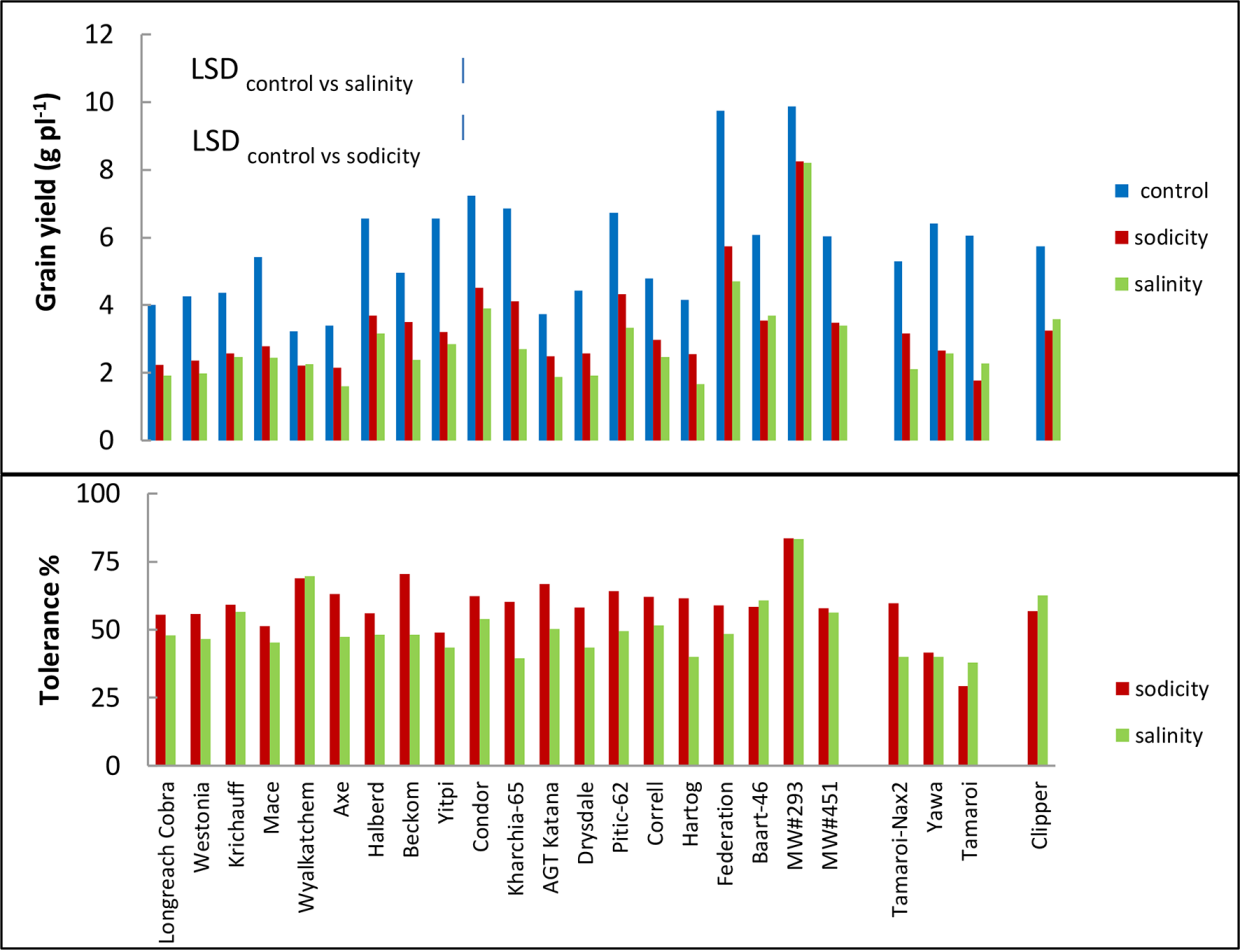
Best linear unbiased estimates for grain yield, and tolerance (grain yield under sodicity or salinity as a percentage of grain yield under control) of 20 bread wheat entries (*Triticum aestivum* L.), three durum wheat entries (*Triticum turgidum* subsp durum cv. Tamaroi, Tamaroi-*Nax2* and Yawa) and one barley (*Hordeum vulgare* L. cv. Clipper) in Experiment 3 (n = 4). The vertical bars indicate Least Significant Difference test value at *P =* 0.05 for variety x treatment interaction. Entries are ordered in ascending order of leaf Na^+^ concentration.

Bread wheat entries varied in grain yield under control, sodicity and salinity (3-, 4-, and 5-fold, respectively; **Figure 5**). Axe produced the lowest, while germplasm line MW#293 produced the highest grain yield under all conditions and doubled the grain yield of almost all other entries under salinity and sodicity (**Figure 5**). There was a close correlation between grain yield produced under control and either salinity or sodicity (r = 0.867 and r = 0.927 when comparing control *vs* salinity or control *vs* sodicity) (**Figure 4**). The correlation was even greater when grain yields were compared between salinity and sodicity (r = 0.961, *df =* 18, *P* < 0.01) (**Figure 4**). Depending on wheat entries, tolerance (relative grain yield %) was higher, lower, or similar between salinity and sodicity (**Figure 5**). Similar to water use, the most noteworthy effects were the higher sodicity tolerance in Tamaroi-*Nax2* compared to Tamaroi, and the highest salinity and sodicity tolerance in MW#293 (**Figure 5**). There was a modest positive correlation between leaf Na^+^ concentration and salinity or sodicity tolerance (**Figure 4**; *df =* 18, *P* < 0.05; r = 0.475 and r = 0.463 for sodicity and salinity respectively). However, this relationship was greatly influenced by the inclusion of MW#293. When MW#293 was omitted from the analyses, there were no correlations.

### Experiment 4. Grain Yield, Leaf Element Concentration, and Expression Profiling of Selected Genes of Interest

**Figure 6:**
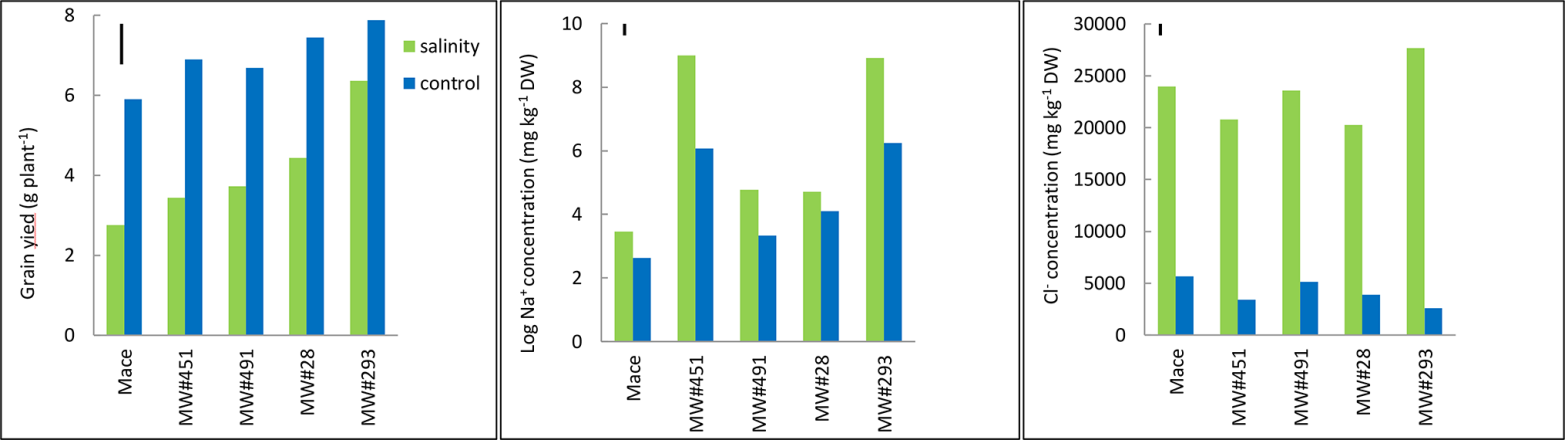
Best linear unbiased estimates for grain yield, and leaf Na^+^ and Cl^-^ concentrations at heading in bread wheat cv. Mace, and two low-Na^+^ (MW#28 and MW#491) and two-high Na^+^ (MW#293 and MW#451) doubled-haploid lines selected from a cross between Mace and high-Na^+^ germplasm W4909 (n = 5). Sodium concentration data were transformed to natural logarithms. The vertical bars indicate Least Significant Difference test value at *P =* 0.05 for variety x treatment interaction. Wheat lines are ordered in ascending order of salinity tolerance (ratio of grain yield under salinity to grain yield under control, expressed as percent).

The five wheat lines varied significantly in grain yield under control and salinity; higher yielding lines under control were also higher yielding under salinity (**Figure 6**). Mace was the lowest yielding, while MW#293 the highest. Salinity tolerance measured as relative grain yield (%) ranged from 47% in Mace to 81% in MW#293. Salinity increased leaf Na^+^ concentrations significantly but mainly in MW#451 and MW#293 (approx. 7,770 and 8,400 mg kg^-1^ DW, respectively), almost two orders of magnitude higher than for Mace, MW#28, and MW#491 (35, 122 and 124 mg kg^-1^ DW, respectively) (**Figure 6**). In agreement with Experiment 3, Cl^-^ concentrations increased significantly under salinity (**Figure 6**). Despite differences across wheat lines and treatments, leaf Ca^2+^, K^+^, and Mg^2+^ concentrations were all above the critical levels for deficiency thus indicating adequate nutrition (**Figure S4**, Reuter and Robinson, 1997).

A number of genes were selected for analysis of gene expression (**Table S2**). These included: Three candidate genes identified by GWAS in Experiment 1, five previously published genes that were differentially expressed under control and salt, and between the parental line W4909 (high-Na^+^ bread wheat) and cv. Chinese Spring (low-Na^+^ bread wheat) (Mott and Wang, 2007), and the *Nax1 (TmHKT1;4-A2)* gene (Tounsi et al., 2016). One of the three candidate genes (*calcium-transporting ATPase*) and *Nax1 gene* had very low levels of expression, data not shown, while two candidate genes (*Na^+^/H^+^* *antiporter NhaB* and *Aquaporin TIF1-4*) were differentially expressed between wheat lines and treatments. *Na^+^/H^+^* *antiporter NhaB* was highly expressed in low-Na^+^ wheat lines Mace, MW#28, and MW#491, while very low expression levels were observed in high-Na^+^ wheat lines MW#293 and MW#451 (**Figure S5**). Wheat lines showed similar expression levels under control and salinity, with the notable exception of MW#491 showing higher expression under salinity (**Figure S5**). *Aquaporin TIF1-4* levels were higher in wheat line Mace than the other four wheat lines, and levels varied depending on treatments; lower (Mace and MW#451), higher (MW#293), or no change (MW#28 and MW#451) under salinity (**Figure S5**). Of the five previously reported genes, four (*Na^+^/H^+^* *antiportersNHX1* and *NHX2*, *putativehigh affinity potassium transporter*, and *vacuolar pyrophosphatase [TaAVP1] similar to AVP1*) were differentially expressed between wheat lines and treatments (**Figure S5**). The main effects were (i) higher expression of *putative high-affinity potassium transporter* and *NHX2* in MW#293 and (ii) higher expression of *NHX1* in MW#451 under salinity, and (iii) lower expression of AVP1 like gene in MW#451 and MW#293 under both control and salinity (**Figure S5**). Moreover, the probe set Ta.22954.1.S1_at showed very low levels of expression in the present study (data not shown); in contrast to an 8-fold higher expression in high-Na^+^ bread wheat germplasm line W4909 (parental line of MW#293) compared with low-Na^+^ Chinese Spring bread wheat under salinity (**Table S2** in Mott andWang 2007).

## Discussion

### Sodium Exclusion in Bread Wheat and Its Relationship With Salinity and Sodicity Tolerance as Measured by a Novel Screening Method

Our results, along with other studies (e.g. Paull et al., 1994), demonstrate that whilst there is genetic variation for Na^+^ concentration in modern bread wheat (n = 98; **Table S3**, **Figure 1**), most wheats contain relatively low Na^+^ concentrations. In modern bread wheat we found no correlation between leaf Na^+^ concentration and either salinity or sodicity tolerance based on grain yield (n = 18; Experiment 3). In fact, wheat germplasm MW#293 carrying alien introgressions achieved the highest salinity tolerance despite having a 14-fold higher Na^+^ concentration (6,044 mg kg^-1^ DW) than the highest of the naturally occurring bread wheats (cv. Federation, 425 mg kg^-1^ DW) (**Table S1**). Similarly, under sodicity MW#293 had a 7-fold higher Na^+^ concentration (12,939 mg kg^-1^ DW) than the second highest bread wheat cv. Federation (1,651mg kg^-1^ DW), and still had the highest sodicity tolerance (**Figure 5**, **Table 2**). Despite the prevailing opinion that low Na^+^ confers tolerance, the results in Experiment 3 and other studies in wheat, barley, and maize show that low Na^+^ concentration is not necessarily associated with salinity tolerance (Rawson et al., 1988; Alberico and Cramer 1993; Cramer et al., 1994; Glenn et al., 1997; Brini et al., 2007; Genc et al., 2007; Ul Haq et al., 2014; Genc et al., 2016; Setter et al., 2016). This suggests that additional mechanisms (tissue tolerance/osmotic adjustment) need to be considered in order to breed salinity tolerant bread wheat.

### Effects of *Nax1* and *Nax2* on Salinity and Sodicity Tolerance in Low-Na^+^ Bread Wheat Cv. Westonia

Our results confirm that Westonia-*Nax1* and Westonia-*Nax2* lines were lower in leaf Na^+^ concentration compared to Westonia, and showed slightly higher but non-significant grain yield increase at moderate salinity (50 mM NaCl) and low sodicity (2 g kg^-1^ Na^+^-humate) (**Figure 3**, **Tables S7** and **S8**). However, compared to high-Na^+^ bread wheat Baart-46, Na^+^ concentrations of Westonia and Nax lines were low, and hence small differences in Na^+^ concentration between Westonia and the Nax lines are unlikely to make a difference to grain yield (**Figure 3**, **Tables S7** and **S8**). This supposition is supported by two lines of evidence: In Experiment 2, Baart-46 had much higher Na^+^ concentration but yielded higher than the three Westonia lines at all levels of salinity and sodicity. Secondly, in saline field trials, only one Westonia-Nax2 line (5924) yielded higher (11%) in 2009 but lower (6.5%) in 2011 than Westonia, while the other four Westonia-Nax lines were, on average, no different to Westonia (**Tables S1** and **S3**; Setter et al., 2016). The results indicate that transferring *Nax1* and *Nax2* genes into an already efficient Na^+^ excluding bread wheat confers little, if any, improvement in overall salinity tolerance. Unlike low-Na^+^ bread wheat, when the *Nax2* gene was introduced into high-Na^+^ durum wheat cv. Tamaroi, a significant yield increase was reported under salinity in the field (Munns et al., 2012) and under sodicity in the growth room (Genc et al., 2016). The differences between the Na^+^ excluding abilities of bread and durum wheats are attributed to modern bread wheats possessing homologs of the Na^+^ exclusion genes *Nax1* and *Nax2* and/or other Na^+^ exclusion genes (Byrt et al., 2007; cf. Genc et al., 2010; Hussain et al., 2017; Oyiga et al., 2018), while durum wheats are thought to lack such genes. Hence, the introduction of Nax type genes is more useful in durum wheat backgrounds.

### SNPs and Candidate Genes for Na^+^ Accumulation, and Their Relationships With Salinity Tolerance

Of the nine SNPs significantly associated with leaf Na^+^ concentration, seven were mapped to chromosomes 2A, 2B, 2D, 4B, 4D, 5B, and 7A, while the rest could not be assigned to a particular chromosome (**Table S4**), hence there is limited discussion with published studies. Using the latest available Chinese Spring Reference Genome (IWGSC RefSeq v1.0), we compared the physical position of previously reported SNPs (Oyiga et al., 2018) to the seven mapped SNPs in the present study. It appears that all seven SNPs are novel. Four candidate genes with putative functions in the regulation of Na^+^ concentration were identified in close physical location to these significant SNPs, of which two were significantly different between treatments and wheat lines in the gene expression study (**Table S2**, **Figure S5**).

Some of the genes described in this study (*NHX1* and *NHX2*, Ta*AVP1*, putative high-affinity potassium transporter) have been previously reported (Mott and Wang, 2007), while others were identified for the first time (**Table S2**). Contrary to no differences in Mott and Wang (2007), *NHX1* and *NHX2* expression levels varied between treatments and wheat lines (**Figure S5**). Like Adem et al. (2017), we found no correlation between expression levels of *NHX* genes and grain yield. However, other studies have found that increased expression of *NHX* genes can enhance growth under saline conditions (Apse et al., 1999; Zhang et al., 2001; Bayat et al., 2011). Non-significant expression levels observed for vacuolar pyrophosphatase gene were in contrast to findings of Mott and Wang (2007), who reported higher expression in high-Na^+^ germplasm line W4909 than low-Na^+^ Chinese Spring bread wheat under control and salinity, but much greater expression levels under control. In transgenic barley, *AVP* expression was correlated with shoot biomass and grain yield (Schilling et al., 2014), while in our study there was no evidence for this. Putative high-affinity transporter, a candidate gene for osmoregulation (Mott and Wang, 2007), had higher expression under salinity in MW#293 only (**Figure S5**), and this was in contrast to higher expression levels in low Na^+^ Chinese Spring bread wheat than in high Na^+^ germplasm W4909 in Mott and Wang (2007). In addition, candidate genes were identified that showed similarity to an *Na^+^/H^+^* *antiporter NhaB*, aquaporin *TIF1-4*, and aquaporin *PIP2*. In *Escherichia coli*, *NhaB* was one of the Na^+^/H^+^ antiporters identified and characterized as a sodium pump (Pinner et al., 1992). Although this gene is uncharacterised in plants, its similarity to a well-characterised Na^+^-H^+^ exchanger family gene, *NHX7* (*SOS1*) (Chanroj et al., 2012), indicates that it could be involved in limiting root uptake of Na^+^ and/or expelling Na^+^ from the leaf tissue. It is possible that in high Na^+^ lines alien introgression has contributed an alternative allele or null at this locus, or altered the expression of this gene resulting in higher leaf Na^+^. As a consequence, this could contribute to tissue tolerance/osmotic adjustment in the plant and improve salt tolerance. As *Na^+^/H^+^* *antiporter NhaB* is approximately 8.4 Mb away from the location of *Kna1* (candidate gene *TaHKT1;5-D* and homologous to *Nax2* gene) (TraesCS4D01G361300; 507,965,585-507,967,671), it could represent a novel candidate for Na^+^ exclusion in wheat. However, its expression levels did not correlate with grain yield in the present study.

Aquaporins, membrane channel proteins, are largely known for their roles in water uptake, and are also involved in the transport of small neutral solutes, gasses, and metal ion (Afzal et al., 2016). Aquaporins in higher plants are classified into five subfamilies; plasma membrane intrinsic proteins (PIPs), tonoplast intrinsic proteins (TIPs), nodulin-26 like intrinsic proteins (NIPs), small basic intrinsic proteins (SIPs), and X intrinsic proteins/uncharacterized-intrinsic proteins (XIPs) (references in Kapilan et al., 2018). Overexpression of the *TIP* genes were reported to increase salt and drought tolerance in *Panax ginseng* and *Jatropha curcas* (Peng et al., 2007; Khan et al., 2015). Similarly, PIPs were shown to be involved in salt and osmotic stress response in barley (Alavilli et al., 2016) and durum wheat (Ayadi et al., 2019). In the present study, although aquaporin *TIF1-4* gene expression was higher in salinity tolerant high-Na line MW#293 under salinity, expression levels did not always correlate with leaf Na^+^ or grain yield.

The differences in gene expression between the present study and previous studies could be attributable to differences in the germplasm used and experimental variables. Analysis of the candidate genes identified across a tissue and time series would provide further information regarding spatial and temporal regulation that would assist with correlation to proposed function in salinity tolerance.

### Potential of Tissue Tolerance/Osmotic Adjustment for Further Improvement of Salinity Tolerance in Bread Wheat

Tissue tolerance (the ability of an organ to maintain function in the presence of elevated tissue Na^+^ and Cl^-^ concentrations) and osmotic adjustment (maintaining turgor by accumulating inorganic ions (mainly Na^+^, K^+^, Ca^2+^, and Cl^-^), organic acids, carbohydrates, and amino acids), are regarded as two of the three main mechanisms of salinity tolerance in plants (Munns and Tester, 2008). These two mechanisms are considered inseparable from one another (Munns et al., 2016), and less is known about their physiological and genetic aspects as compared to the Na^+^ exclusion mechanism. It has been reported that more tolerant species accumulate Na^+^ and Cl^-^ in their roots and shoots similar to the external solution, thereby providing energy-efficient osmotic adjustment (Munns et al, 2016). As Na^+^ concentration is generally much lower in bread wheat than durum wheat, it is less efficient in adjusting its osmotic potential *via* inorganic ions (Na^+^, K^+^, and Cl^-^), and relies heavily on organic solutes instead (Cuin et al., 2009), which is energetically costly (Raven, 1985). There is now an opportunity to build the energy-efficient osmotic adjustment mechanism into modern bread wheat. In the present study, GWAS identified alleles of candidate genes from MW#451 and MW#293 associated with high leaf Na^+^ (**Tables S4** and **S5**). This, together with gene expression data here (**Figure S5**) and elsewhere (Mott and Wang, 2007) indicate that these genes could be involved in tissue tolerance of wheat germplasm MW#293. Higher relative growth rates in MW#293 under salinity also points to better osmotic adjustment in this line. Taken together, tissue tolerance and osmotic adjustment are likely to contribute to higher salinity of MW#293.

### A Novel Wheat Germplasm (MW#293) for Development of Future Salinity/Sodicity Tolerant Bread Wheat

MW#293 was derived from an earlier bread wheat germplasm line (W4909) developed by Richard Wang and his colleagues (Wang et al., 2003). W4909 is a product of three species [*Triticum aestivum* cv. Chinese Spring, *Aegilops speltoides*, and *Thinopyrum junceum* (sea wheatgrass)], and its ability to accumulate very high Na^+^ sodium has been demonstrated in independent studies (Wang et al., 2003; Genc et al., 2007; Mott and Wang, 2007). However, its salt tolerance is debatable as studies so far have produced variable results (Wang et al., 2003; Genc et al., 2007; Mott and Wang, 2007). In addition, the potential of high Na^+^ as a source of osmotic adjustment/tissue tolerance in a widely adapted and high yielding bread wheat has not been realized.

**Figure 7:**
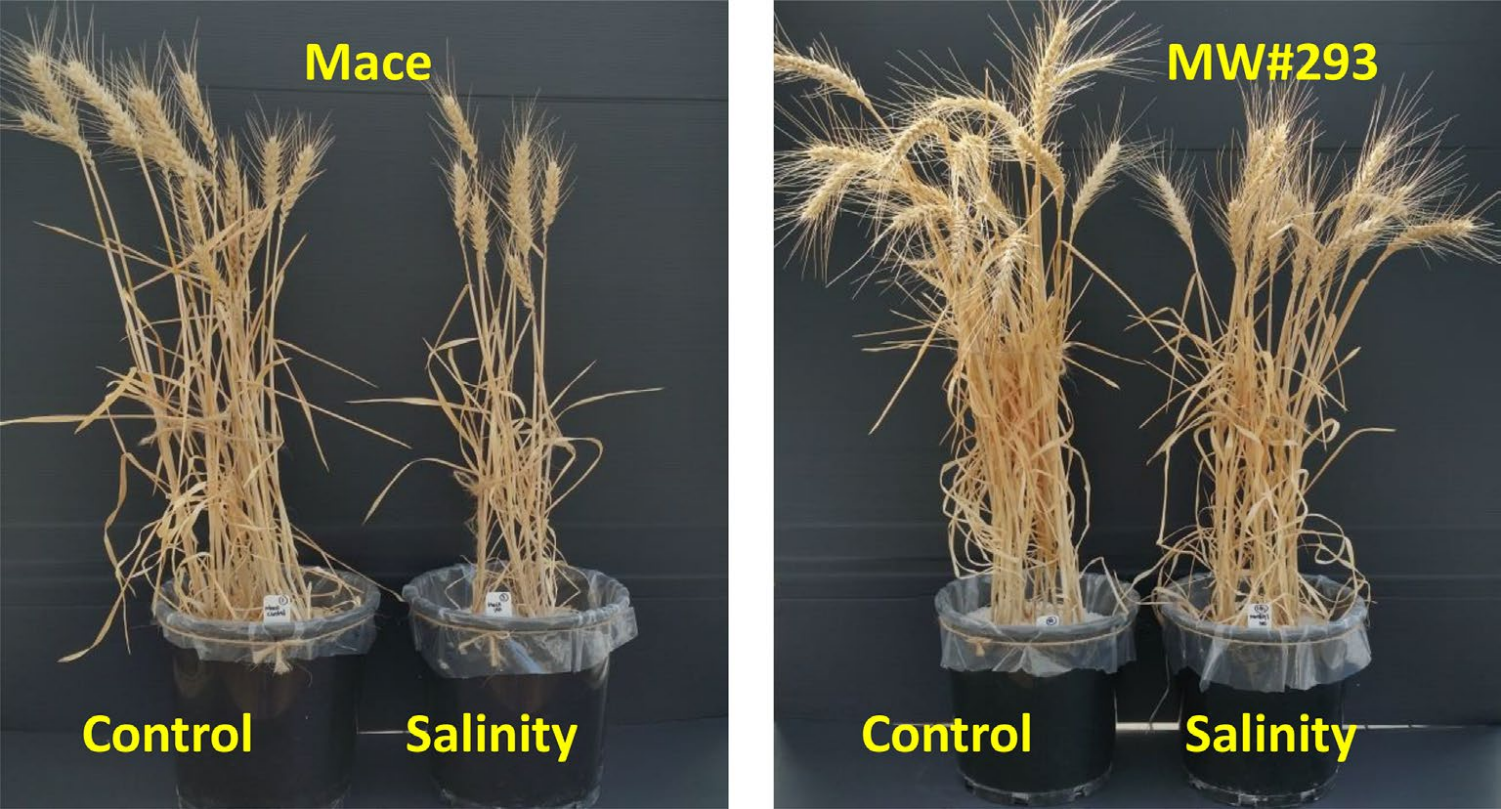
Representative pots of bread wheat (*Triticum aestivum*) cv. Mace and doubled-haploid line MW#293 grown under control and salinity (100 mM NaCl) in Experiment 4.

To introduce salt tolerance gene(s) of W4909 into a commercial bread wheat, we made a cross between a popular Australian bread wheat cv. Mace and W4909, and developed a doubled-haploid population (over 200 lines). As the population segregated for maturity and height markedly, a sub-selection of this population (n = 18), agronomically similar to commercial lines, was grown under control and salinity using the soil assay described in Genc et al. (2016). Of these 18 lines, MW#293 had the highest grain yield under both control and salinity, and doubled the grain yield of Mace under salinity despite having an 86-fold higher leaf Na^+^. When tested with 18 commercial wheats in Experiment 3 (which included Kharchia 65-one of the most sodicity- and salinity-tolerant landraces; Chhipa and Lal, 1985), MW#293 produced the highest grain yield under control, salinity and sodicity, and its grain yield under salinity was three times higher despite 35-100-fold higher leaf Na^+^ concentrations (under sodicity and salinity) than cv. Mace (**Table 2**). Mujeeb-Kazi and colleagues (2019, p. 52) make the important point that breeding wheat solely for salinity tolerance at the cost of yield loss in nonsaline soils is unsuitable for farmers: “Breeders need to develop cultivars with high yield potential under both stress and nonstress conditions”, in other words *vigorous* cultivars. In Experiment 4, MW#293 recorded 200-fold higher leaf Na^+^ concentration and 2-fold higher grain yield than cv. Mace (**Figures 6** and **7**). MW#293 also had the highest growth rates under salinity and sodicity (**Table S9**). These data suggest that MW#293 may have the ability to efficiently assimilate and sequester Na^+^ levels that can support high growth rates (Glenn et al., 1997). To our knowledge, such high Na^+^ accumulation together with high grain yield/growth rate in bread wheat has not been previously reported. This represents a new paradigm in breeding for salinity tolerance.

## Conclusions

- Despite much higher leaf Na^+^ concentration, bread wheat germplasm MW#293 had higher grain yield under salinity and sodicity, in absolute and relative terms, than the other bread wheat entries tested.
- Despite a 10–14 fold variation in leaf Na^+^ concentration in modern bread wheats, there were no correlations between leaf Na^+^ concentration and either salinity or sodicity tolerance, thus demonstrating the limits of using leaf Na^+^ concentration alone as a selection parameter for salinity/sodicity tolerance.
- As modern bread wheats have an excellent Na^+^ exclusion ability, further investment in the Na^+^ exclusion mechanism is unlikely to improve sodicity/salinity tolerance significantly. Future efforts should focus on osmotic adjustment/tissue tolerance mechanisms.
- Genome-wide association mapping revealed novel genes associated with high Na^+^ accumulation, which may be involved in osmotic adjustment/tissue tolerance.

The salinity and sodicity tolerant germplasm MW#293 provides an opportunity for the development of future salinity/sodicity tolerant bread wheat.

## Data Availability Statement

All datasets for this study are included in the manuscript/**Supplementary Files**.

## Author Contributions

YG, MA, KO, and TS were involved in experimental design and the conceptualization of the project. YG and GL carried out phenotyping and drafted the manuscript. JT performed statistical analysis of the data and drafted the manuscript. YL performed GWAS and drafted the manuscript. JC performed gene expression and drafted the manuscript. All authors read and approved the final manuscript.

## Funding

This work was supported by South Australian Research and Development Institute, The Waite Research Institute, The Yitpi Foundation, The Grains Research and Development Corporation, and The University of Adelaide.

## Conflict of Interest

The authors declare that the research was conducted in the absence of any commercial or financial relationships that could be construed as a potential conflict of interest.
