# Supplementary material for: Bread Wheat With High Salinity and Sodicity Tolerance
Source: Front Plant Sci. 2019 Oct 22;10:1280. doi: 10.3389/fpls.2019.01280 (PMC6817574; doi:10.3389/fpls.2019.01280)
Supplement: Supplementary file 1 [file Table_1.docx]

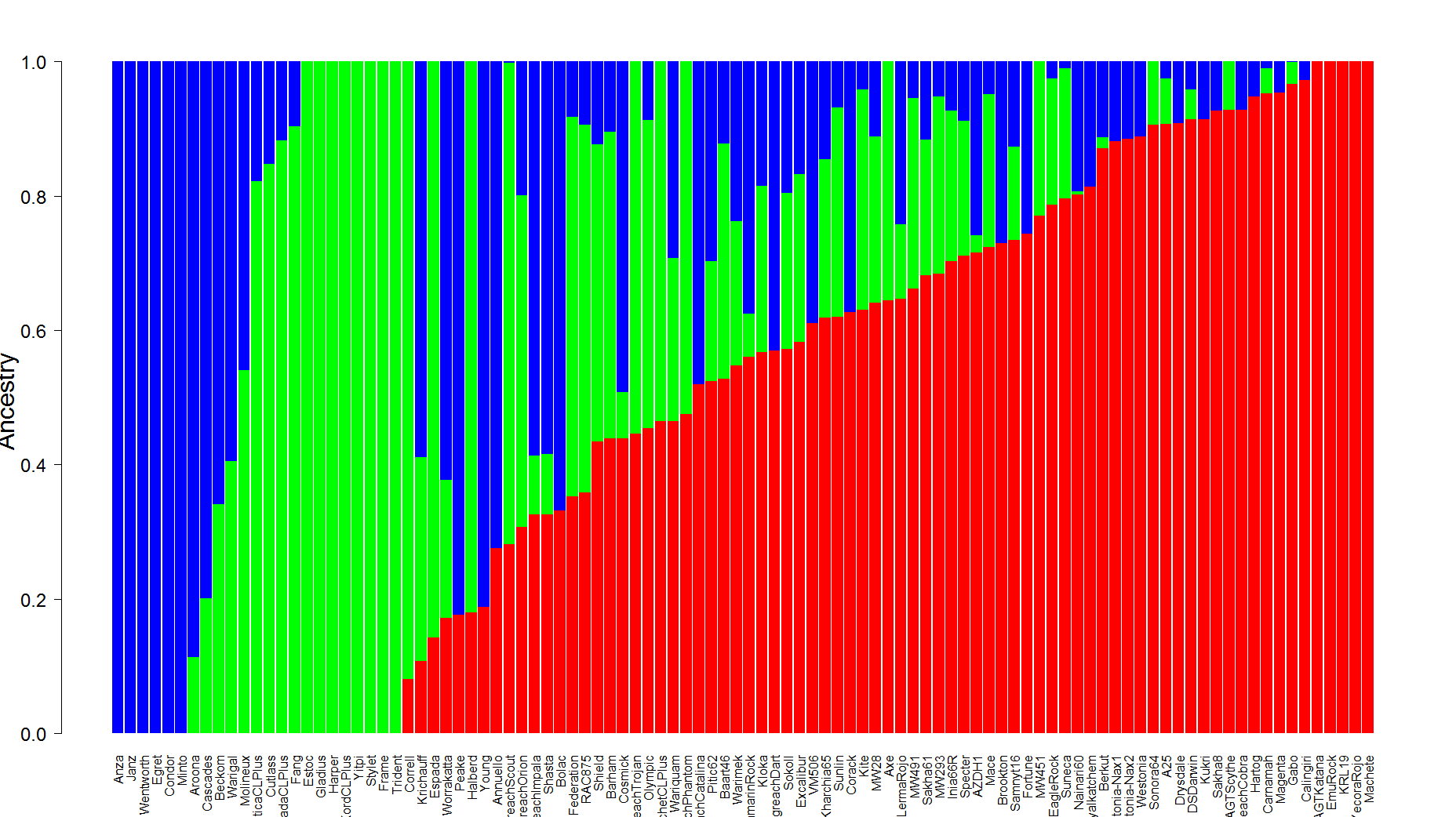


**Figure S1** Population structure based on 41,036 SNP markers (MAF>0.05). Each genotype is represented by a horizontal bar partitioned into *k* coloured segments that represent the genotype’s estimated membership fractions. The most likely number of clusters in this population is *k*=3.

**Salinity**

**Sodicity**


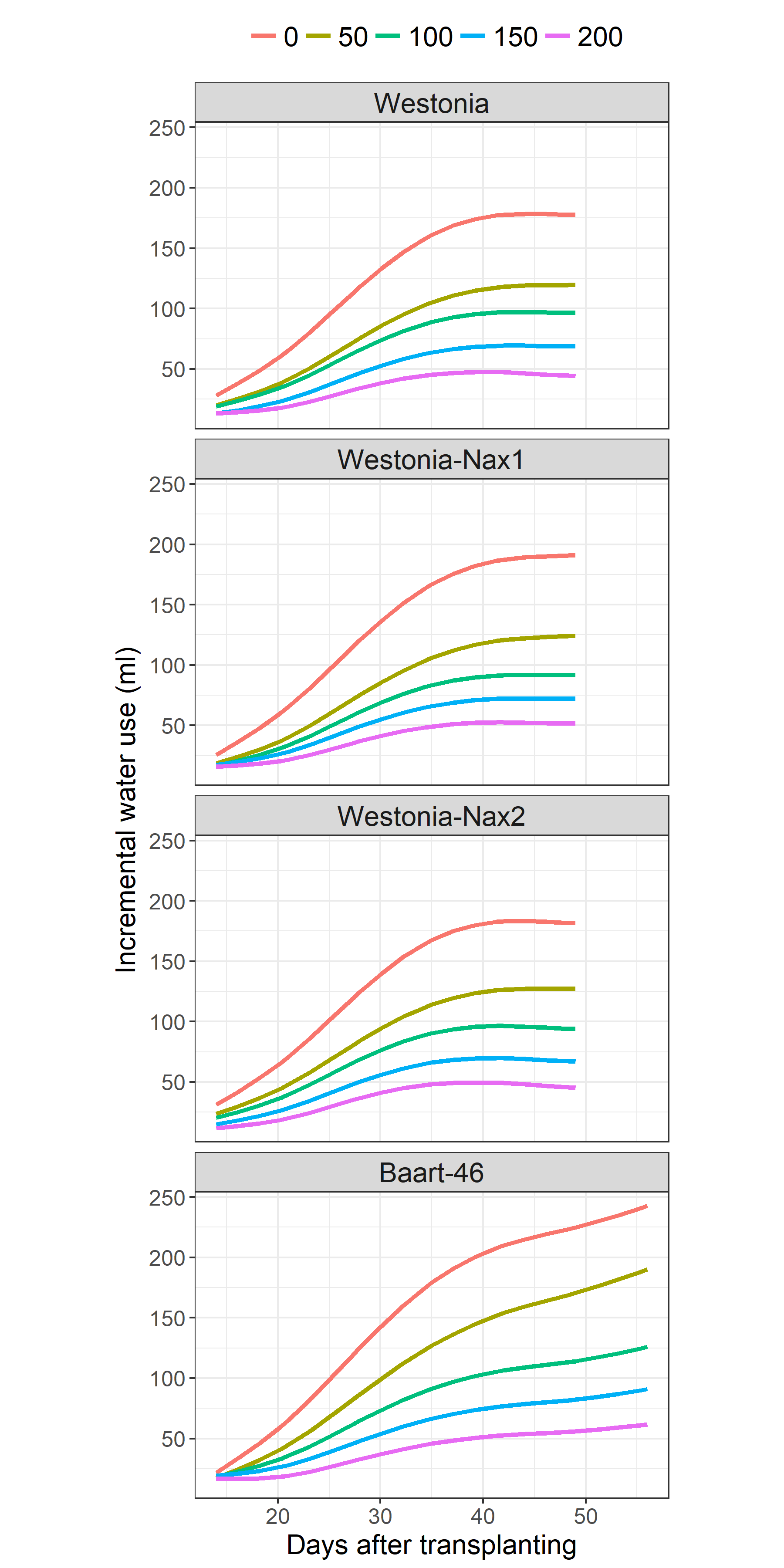

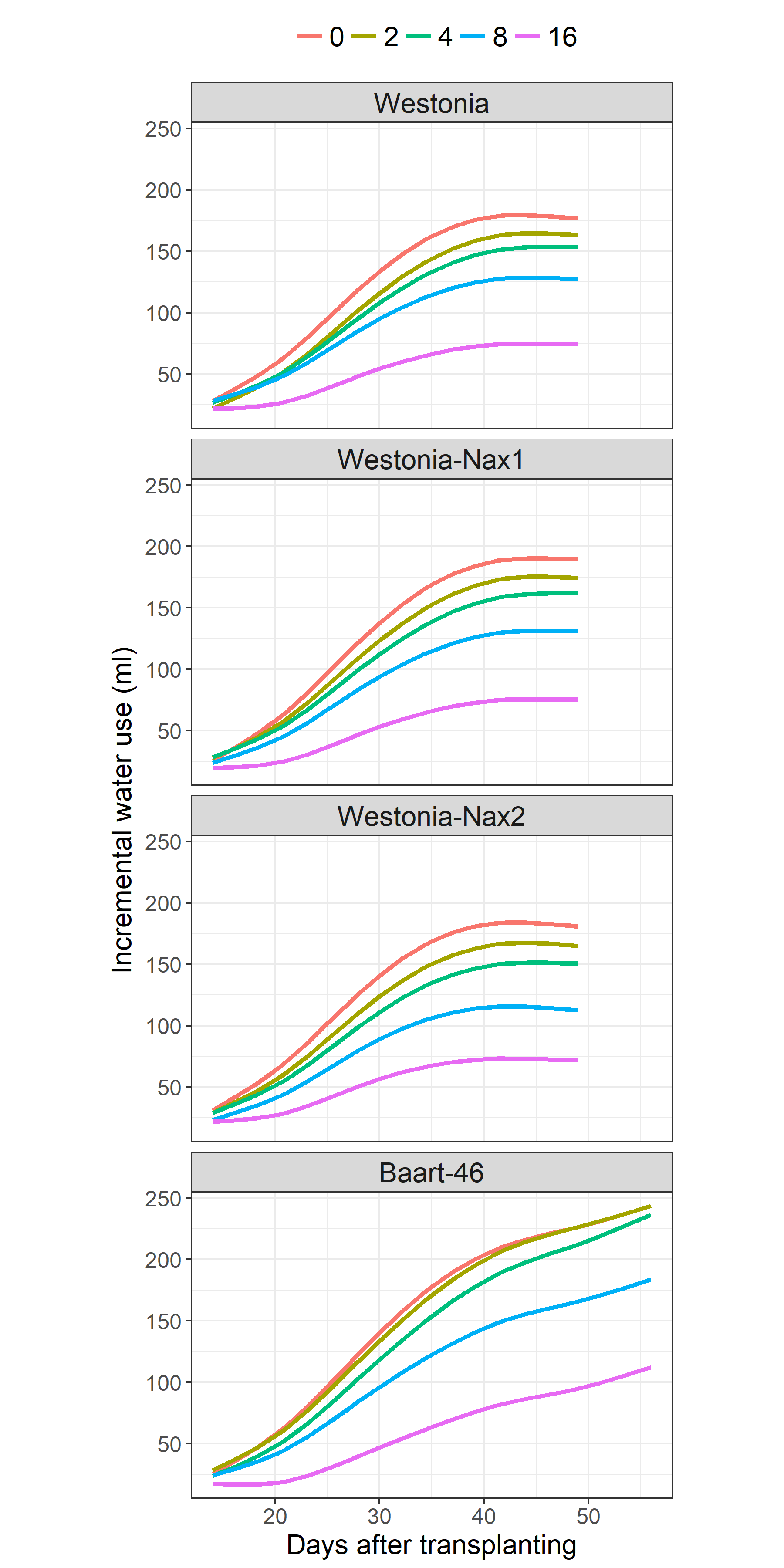


**Figure S2** Incremental water use (weekly) curves until heading in Westonia, Westonia-*Nax1*, Westonia-*Nax2* and Baart-46 under different levels of salinity (mM NaCl, left panels) and sodicity (g kg^-1^ Na^+-^humate, right panels) in Experiment 2 (n=4). Estimated slopes of the linear component of these curves are presented in **Table S6**.


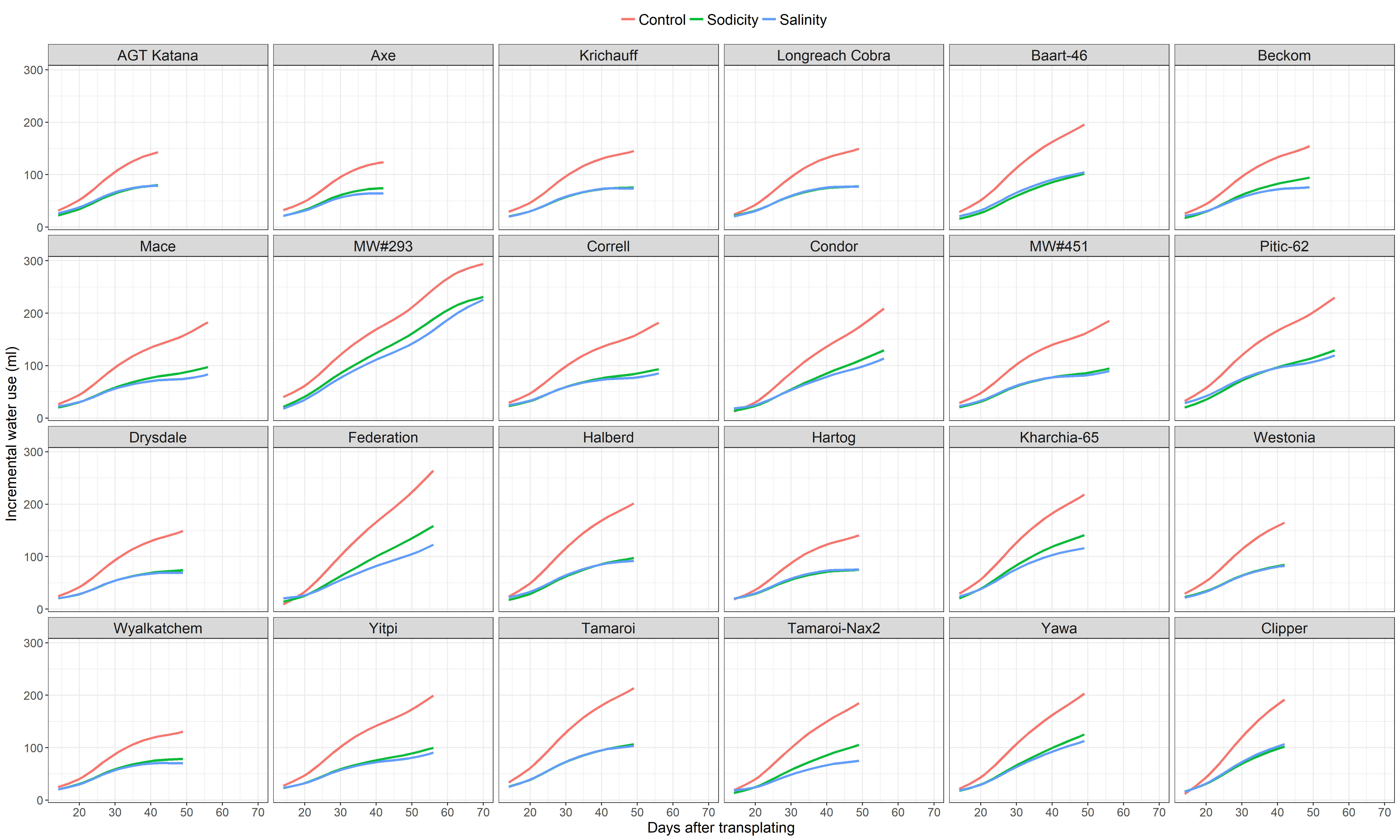


**Figure S3** Incremental water use curves until heading in 20 bread wheat entries (*Triticum aestivum L*.), three durum wheat entries (*Triticum turgidum* subsp durum cv. Tamaroi, Tamaroi-*Nax2* and Yawa) and one barley (*Hordeum vulgare* L. cv. Clipper) under control, salinity (100 mM NaCl) and sodicity (8 g kg^-1^ Na^+^-humate) in Experiment 3 (n=4). Estimated slopes of the linear component of these curves are presented in **Table S9**.


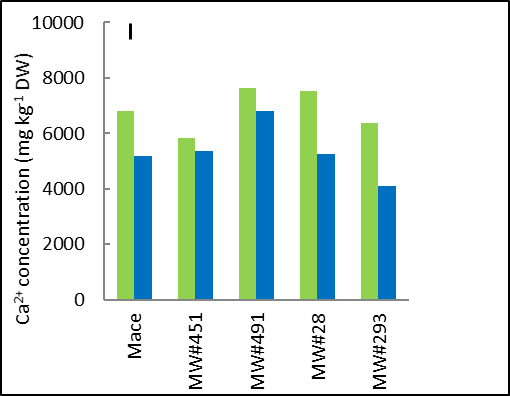

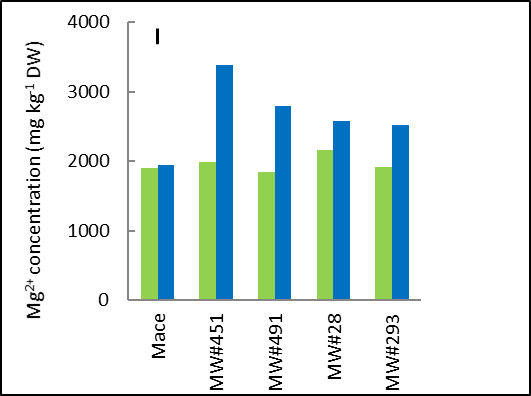

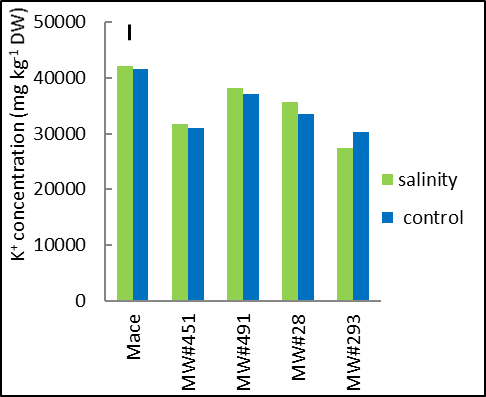


**Figure S4** Best linear unbiased estimates for leaf K^+^, Ca^2+^ and Mg^2+^ concentrations at heading in bread wheat (*Triticum aestivum* L.) cv. Mace, and two low-Na^+^ (MW#28 and MW#491) and two high-Na^+^ (MW#293 and MW#451) doubled-haploid lines selected from a cross between Mace and high-Na^+^ germplasm W4909 in Experiment 4 (n=5). The vertical bars indicate Least Significant Difference test values at *P*=0.05 for variety x treatment interactions. Wheat lines are ordered in ascending order of salinity tolerance (ratio of grain yield under salinity to grain yield under control, expressed as percent).


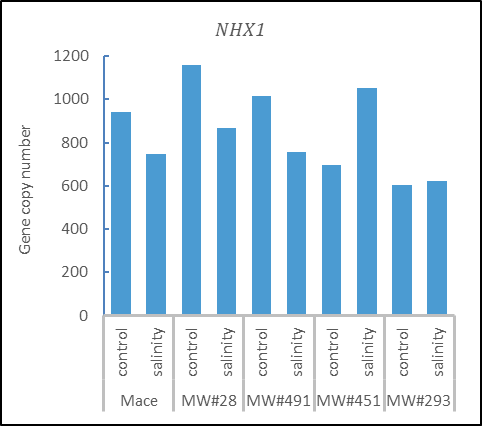

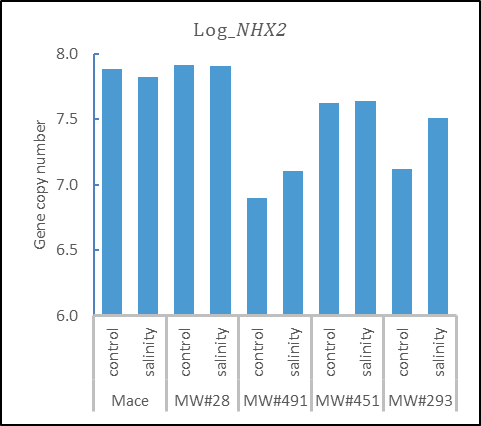

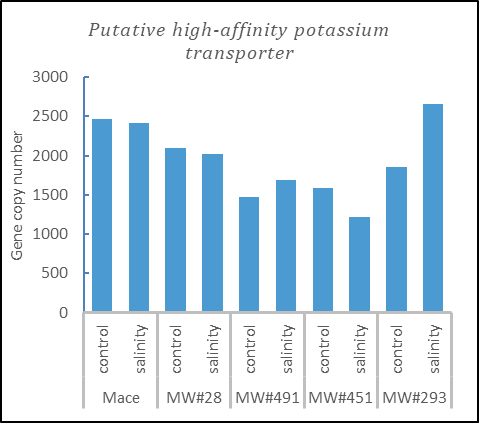

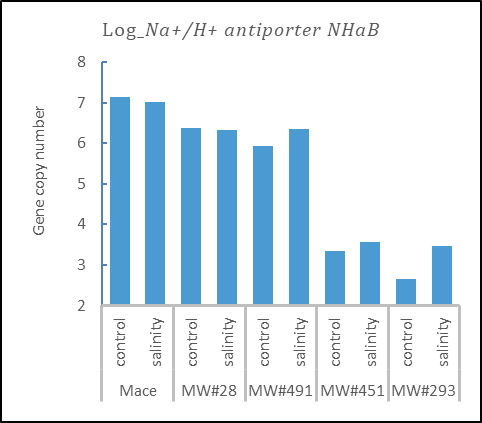

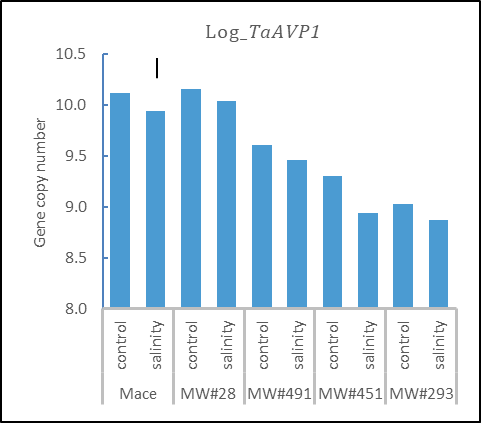

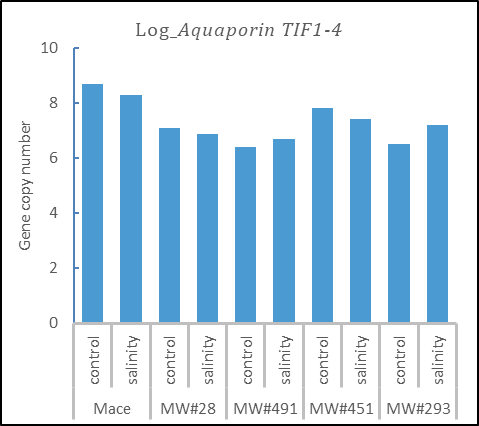


**Figure S5** Best linear unbiased estimates (BLUEs) for candidate genes (copy number ng^-1^ mRNA) identified by GWAS in Experiment 1 (*Aquaporin TIF1-4* and *Na^+^/H^+^ antiporter NHaB*) and known genes (*NHX1, NHX2, Putative high-affinity potassium transporter* and *TaAVP1)* in penultimate leaves in low-Na^+^ bread wheat (*Triticum aestivum* L.) cv. Mace, and two low-Na^+^ (MW#28 and MW#491) and two high-Na^+^ (MW#293 and MW#451) doubled-haploid lines selected from a cross between wheat cv. Mace and a high-Na^+^ line W4909 under control and salinity (100 mM NaCl) in Experiment 4 (n=9). The vertical bars indicate Least Significant Difference test value at *P*=0.05 for variety x treatment interaction.

| **Table S1.** Name**,** year of registration, origin, species, pedigree and the source of entries used in Experiment 1. Most wheat pedigree data were compiled from http://www.wheatpedigree.net/ | | | | | | |  |
| --- | --- | --- | --- | --- | --- | --- | --- |
|  | Name | Year of registration | Origin | Species | Pedigree | Source |  |
| 1 | A25 | breeding line | Australia | bread | Krichauff/Ceta/Ae.squarrosa(895), a DH line from the Krichauff * 29663 cross; HW768*A25 | South Australian Research and Development Institute |  |
| 2 | **AGT Katana** | 2009 | Australia | bread | KUKRI/TAMMIN | Australian Grains Genebank |  |
| 3 | **AGT Scythe** | 2004 | Australia | bread | CO-4080-109/CO-3749-009 | Australian Grains Genebank |  |
| 4 | Annuello | 2001 | Australia | bread | AVON(SIB)/TM-56(VF-665)//JANZ | Australian Grains Genebank |  |
| 5 | Anza | 1971 | USA | bread | LERMA-ROJO-64//NORIN-10/BREVOR/3/3*ANDES-ENANO | Australian Grains Genebank |  |
| 6 | Aroona | 1981 | Australia | bread | WW-15/RAVEN | University of Adelaide |  |
| 7 | **Axe** | 2007 | Australia | bread | (DH)RAC-875//EXCALIBUR/KUKRI/3/RAC-875//EXCALIBUR/KUKRI | Australian Grains Genebank |  |
| 8 | AZDH1 | breeding line | India | bread | AZDH1 =KRL1-4*HD2329 | South Australian Research and Development Institute |  |
| 9 | Baart-46 | 1948 | USA | bread | BAART-38(S)/2*BAART | Australian Grains Genebank |  |
| 10 | **Barham** | 2007 | Australia | bread | BOWIE,AUS//BERSEE/3*BINDAWARRA-126937/3/BOWIE | Australian Grains Genebank |  |
| 11 | **Beckom** | 2015 | Australia | bread | was selected from a cross involving Annuello, Stylet and Young. | LongReach Plant Breeders |  |
| 12 | Berkut | 2002 | Mexico | bread | IRENA/BAVIACORA-M-92//PASTOR; IRENE/BABAX//PASTOR | South Australian Research and Development Institute |  |
| 13 | **Bolac** | 2006 | Australia | bread | NESSER/2*VI-252 | Australian Grains Genebank |  |
| 14 | Brookton | 1997 | Australia | bread | 76-W-595//CRANBROOK/TORRES; TORRES/CRANBROOK//76-W-596/CRANBROOK | LongReach Plant Breeders |  |
| 15 | Calingiri | 1997 | Australia | bread | [CHINO/KULIN//REEVES](http://www.wheatpedigree.net/refer/ajaxShow/2854) | LongReach Plant Breeders |  |
| 16 | Carnamah | 1996 | Australia | bread | BOLSENA-1-CH(RAC-529)/77-W-660; RAC-529-911/77-W-660 | LongReach Plant Breeders |  |
| 17 | Cascades | 1994 | Australia | bread | AROONA*3//(AUSEN-VII-95)TADORNA/INIA-66; AROONA*3//AUSEN-VII-95/QUALSET-601-20 | University of Adelaide |  |
| 18 | Condor | 1973 | Australia | bread | WW-80/2*WW-15 | Australian Grains Genebank |  |
| 19 | **Corack** | 2011 | Australia | bread | WYALKATCHEM/SILVERSTAR//WYALKATCHEM | Australian Grains Genebank |  |
| 20 | **Correll** | 2006 | Australia | bread | RAC-875/YITPI | Australian Grains Genebank |  |
| 21 | **Cosmick** | 2014 | Australia | bread | is a derivative of WA variety, EGA Bonnie Rock and Queensland variety Strzelecki. | Australian Grains Genebank |  |
| 22 | **Cutlass** | 2015 | Australia | bread | RAPIER/BOUNTY | LongReach Plant Breeders |  |
| 23 | Drysdale | 2001 | Australia | bread | HARTOG*3/QUARRION | Australian Grains Genebank |  |
| 24 | **DS Darwin** | 2015 | Australia | bread | CFR00-18/Rubric | South Australian Research and Development Institute |  |
| 25 | Ega-Eagle-Rock | 2004 | Australia | bread | BLADE,AUS*2/SUNELG[3793]; SUNELG*2/BLADE | LongReach Plant Breeders |  |
| 26 | Egret | 1973 | Australia | bread | HERON/2*WW-15 | Australian Grains Genebank |  |
| 27 | **Emu Rock** | 2011 | Australia | bread | is a derivative of parents Westonia, Kukri, Perenjori and Ajana. | Australian Grains Genebank |  |
| 28 | **Espada** | 2008 | Australia | bread | (DH)RAC-875/KRICHAUFF//EXCALIBUR/KUKRI/3/RAC-875/KRICHAUFF/4/RAC-875//EXCALIBUR/KUKRI | Australian Grains Genebank |  |
| 29 | **Estoc** | 2010 | Australia | bread | STYLET//(CO-6143)VM-931/RAC-935 | Australian Grains Genebank |  |
| 30 | Excalibur | 1990 | Australia | bread | [RAC-177(Sr26)/UNICULM-492//RAC-311-S](http://www.wheatpedigree.net/refer/ajaxShow/1326) | LongReach Plant Breeders |  |
| 31 | Fang | 2008 | Australia | bread | [(DH)ANNUELLO/2*STYLET[3798];](http://www.wheatpedigree.net/refer/ajaxShow/3798) | Australian Grains Genebank |  |
| 32 | Federation | 1901 | Australia | bread | YANDILLA/PURPLE-STRAW; PURPLE-STRAW/YANDILLA; YANDILLA-KING/PURPLE-STRAW; PURPLESTRAW-14-A/YANDILLA | Australian Grains Genebank |  |
| 33 | Fortune | 2008 | Australia | bread | CALINGIRI/(95-Y-210)CALINGIRI//CALINGIRI/WORRAKATTA | LongReach Plant Breeders |  |
| 34 | Frame | 1997 | Australia | bread | MOLINEUX/3*DAGGER | Australian Grains Genebank |  |
| 35 | Gabo | 1942 | Australia | bread | BOBIN(S)/(TR.DR)GAZA//(S)BOBIN | Australian Grains Genebank |  |
| 36 | **Gladius** | 2006 | Australia | bread | (DH)RAC-875/KRICHAUFF//EXCALIBUR/KUKRI/3/RAC-875/KRICHAUFF/4/RAC-875//EXCALIBUR/KUKRI | Australian Grains Genebank |  |
| 37 | **Grenade ^CL Plus^** | 2012 | Australia | bread | GLADIUS/4/RAC/1268*2/3/JANZ*2//WILG-4/11-A | Australian Grains Genebank |  |
| 38 | Halberd | 1969 | Australia | bread | SCIMITAR/KENYA-C-6042//BOBIN/3/INSIGNIA-49 | LongReach Plant Breeders |  |
| 39 | **Harper** | 2013 | Australia | bread | FULTZ/MEDITERRANEAN | Australian Grains Genebank |  |
| 40 | Hartog | 1982 | Australia | bread | VICAM-71//CIANO-67(SIB)/SIETE-CERROS-66/3/KALYANSONA/BLUEBIRD | Australian Grains Genebank |  |
| 41 | **Hatchet ^CL Plus^** | 2015 | Australia | bread | derived from the very early maturing variety Axe. | LongReach Plant Breeders |  |
| 42 | Inia 66R | 1970 | USA | bread | a selection from Lerma Rojo 64' X 'Sonora 64 | Australian Grains Genebank |  |
| 43 | Janz | 1989 | Australia | bread | 3-AG-3/4*CONDOR//COOK | Australian Grains Genebank |  |
| 44 | **Justica ^CL Plus^** | 2011 | Australia | bread | GLADIUS/4/RAC-1268*2/3/JANZ*2//WILG-4/11-A | Australian Grains Genebank |  |
| 45 | Kharchia-65 | 1971 | India | bread | EG-593/KHARCHIA; KHARCHIA-LOCAL*5/EG-953; UP-2441/K-9330 | South Australian Research and Development Institute |  |
| 46 | Kite | 1973 | Australia | bread | NORIN-10/BREVOR(SEL.14)//4*EUREKA-2/3/T-A/3*FALCON/4/THATCHER/AG.EL(T-A)//4*FALCON/5/THATCHER/AG.EL(T-A)//5*FALCON | Australian Grains Genebank |  |
| 47 | Kloka | 1965 | Deutschland | bread | RUMKERS-ERLI/KLOKA-309 | Australian Grains Genebank |  |
| 48 | **Kord ^CL Plus^** | 2011 | Australia | bread | GLADIUS*2/4/FRAME//WILG-4/11-A/3*SUNMIST | Australian Grains Genebank |  |
| 49 | Krichauff | 1998 | Australia | bread | WARIQUAM//KLOKA/PITIC-62/3/WARIMEK/HALBERD/4/3-AG-3/AROONA; WARIQUAM//KLOKA/PITIC-62/3/HALBERD/4/3-AG-3/AROONA | University of Adelaide |  |
| 50 | KRL-19 | 1999 | India | bread | PBW-255/KRL-1-4 | South Australian Research and Development Institute |  |
| 51 | Kukri | 1999 | Australia | bread | MADDEN/6*RAC-177//GRAJO/76-ECN-44; SR13*3//76-ECN-44/76-ECN-36; CO-1213/RAC-549; DRP((FRONTANA/KENYA-58//N10B/G55)NAINARI-60)/(TOBARI-66/CIANO-67//TOBARI-66/8156/3/CALIDAD//BLUEBIRD/CIANO-67)/2/MADDEN/6*RAC-177 | Australian Grains Genebank |  |
| 52 | Lerma Rojo-54 | 1954 | Mexico | bread | LERMA-50/YAQUI-48//MARIA-ESCOBAR*2/SUPREMO-211 | Australian Grains Genebank |  |
| 53 | **Longreach Catalina** | 2006 | Australia | bread | VI-184/SILVERSTAR | Australian Grains Genebank |  |
| 54 | **Longreach Cobra** | 2011 | Australia | bread | WESTONIA/W-29 | Australian Grains Genebank |  |
| 55 | **Longreach Dart** | 2012 | Australia | bread | SUNBROOK/JANZ//KUKRI | Australian Grains Genebank |  |
| 56 | **Longreach Impala** | 2011 | Australia | bread | TEAL,AUS/C-93-8//9908 | Australian Grains Genebank |  |
| 57 | **Longreach Orion** | 2010 | Australia | bread | TATIARA/QAL-2000 | Australian Grains Genebank |  |
| 58 | **Longreach Phantom** | 2012 | Australia | bread | YITPI/W-29; SENTINEL-3-R/YITPI | Australian Grains Genebank |  |
| 59 | **Longreach Scout** | 2007 | Australia | bread | SUNSTATE/QH-71-6//YITPI | Australian Grains Genebank |  |
| 60 | **Longreach Trojan** | 2013 | Australia | bread | LPB 00LR000041/Sentinel3R | LongReach Plant Breeders |  |
| 61 | **Mace** | 2007 | Australia | bread | WYALKATCHEM/STYLET//WYALKATCHEM | Australian Grains Genebank |  |
| 62 | Machete | 1985 | Australia | bread | MEC-3/2*GABO(RAC-177)//MADDEN | LongReach Plant Breeders |  |
| 63 | Magenta | 2007 | Australia | bread | CARNAMAH/TAMMIN-18; CARNAMAH/TAMMIN | LongReach Plant Breeders |  |
| 64 | Minto | 1987 | Australia | bread | CONDOR/OLYMPIC//EGRET | Australian Grains Genebank |  |
| 65 | Molineux | 1988 | Australia | bread | PITIC-62/FESTIGUAY//2*WARIGAL | Australian Grains Genebank |  |
| 66 | MW#28 | breeding line | Australia | bread | a DH line from a cross between Mace and a high sodium bread wheat line W4909 | South Australian Research and Development Institute |  |
| 67 | MW#293 | breeding line | Australia | bread | a DH line from a cross between Mace and a high sodium bread wheat line W4909 | South Australian Research and Development Institute |  |
| 68 | MW#451 | breeding line | Australia | bread | a DH line from a cross between Mace and a high sodium bread wheat line W4909 | South Australian Research and Development Institute |  |
| 69 | MW#491 | breeding line | Australia | bread | a DH line from a cross between Mace and a high sodium bread wheat line W4909 | South Australian Research and Development Institute |  |
| 70 | Nainari 60 | 1960 | Mexico | bread | SUPREMO/MENTANA//GABO-55/3/THATCHER/QUERETARO//KENTANA/5/GABO-55 | Australian Grains Genebank |  |
| 71 | Olympic | 1956 | Australia | bread | BALDMIN/QUADRAT | Australian Grains Genebank |  |
| 72 | **Peake** | 2007 | Australia | bread | VN-150/VN-715 | Australian Grains Genebank |  |
| 73 | Pitic-62 | 1962 | Mexico | bread | YAKTANA-54//(SEL.26-1-C)NORIN-10/BREVOR | Australian Grains Genebank |  |
| 74 | RAC875 | breeding line | Australia | bread | [RAC-655/3/Sr21/4*LANCE//4*BAYONET](http://www.wheatpedigree.net/refer/ajaxShow/000) | University of Adelaide |  |
| 75 | Sakha 61 | 1980 | Egypt | bread | [INIA-66/RL-4220//SIETE-CERROS-66/YAQUI-50](http://www.wheatpedigree.net/refer/ajaxShow/3396) | Australian Grains Genebank |  |
| 76 | Sakha-8 | 1976 | Egypt | bread | CIANO-67(SIB)//SONORA-64/KLEIN-RENDIDOR/3/II-8156 | Australian Grains Genebank |  |
| 77 | Samnyt 16 | breeding line | Mexico | bread | could not be established. | South Australian Research and Development Institute |  |
| 78 | Shasta | 1976 | USA | bread | INIA-66/ANZA | Australian Grains Genebank |  |
| 79 | **Shield** | 2012 | Australia | bread | AGT-SCYTHE/CO-7138(CO-7412)//(CO-7413)RAC-1105/CO-7165 | Australian Grains Genebank |  |
| 80 | Sokoll | 2002 | Mexico | bread | [PASTOR/3/ALTAR-84/AE.SQ(TR.TA)//OPATA-M-85](http://www.wheatpedigree.net/refer/ajaxShow/3692) | South Australian Research and Development Institute |  |
| 81 | Sonora-64 | 1964 | Mexico | bread | YAKTANA-54//NORIN-10/BREVOR/3/2*YAQUI-54 | Australian Grains Genebank |  |
| 82 | **Specter** | 2015 | Australia | bread | derived from Mace | LongReach Plant Breeders |  |
| 83 | Stylet | 2001 | Australia | bread | [MOLINEUX/2*TRIDENT](http://www.wheatpedigree.net/refer/ajaxShow/3752) | University of Adelaide |  |
| 84 | Suneca | 1982 | Australia | bread | CIANO-67//SPICA/(AMBER-MUTANT)SONORA-64 | Australian Grains Genebank |  |
| 85 | Sunlin | 1996 | Australia | bread | SUNELG*2//SUNECA*3/VPM-1 | Australian Grains Genebank |  |
| 86 | Tammarin-Rock | 2005 | Australia | bread | KALANNIE/81-Y-970 | LongReach Plant Breeders |  |
| 87 | Trident | 1993 | Australia | bread | VPM-1/5*COOK//4*SPEAR | Australian Grains Genebank |  |
| 88 | VM506 | breeding line | Australia | bread | CIQ/Matong//VF508/3/VF19 | University of Adelaide |  |
| 89 | Warigal | 1978 | Australia | bread | WW-15/RAVEN | Australian Grains Genebank |  |
| 90 | Warimek | 1971 | Australia | bread | MEXICO-120/KODA | Australian Grains Genebank |  |
| 91 | Wariquam | 1971 | Australia | bread | MEXICO-120/QUADRAT | Australian Grains Genebank |  |
| 92 | Wentworth | 2004 | Australia | bread | JANZ*2/VULCAN | Australian Grains Genebank |  |
| 93 | Westonia | 1997 | Australia | bread | CO-1190-203/84-W-127-501 | Commonwealth Scientific and Industrial Research Organisation |  |
| 94 | Westonia-*Nax1* (line 5907) | breeding line | Australia | bread | Westonia with sodium exclusion gene *Nax1* | Commonwealth Scientific and Industrial Research Organisation |  |
| 95 | Westonia-*Nax2* (line 5924) | breeding line | Australia | bread | Westonia with sodium exclusion gene *Nax2* | Commonwealth Scientific and Industrial Research Organisation |  |
| 96 | Worrakatta | 1997 | Australia | bread | [3-AG-3/AROONA/4/HALBERD/WARIMEK//PITIC-62/KLOKA/3/WARIQUAM](http://www.wheatpedigree.net/refer/ajaxShow/2854) | Australian Grains Genebank |  |
| 97 | **Wyalkatchem** | 2001 | Australia | bread | MACHETE/3/(84-W-129-504)GUTHA//JACUP*2/11th-ISEPTON-135 | Australian Grains Genebank |  |
| 98 | Yecora Rojo | 1975 | USA | bread | CIANO-67//SONORA-64/KLEIN-RENDIDOR/3/II-8156; CIANO-67(SIB)//SONORA-64/KLEIN-RENDIDOR/3/II-8156 | Australian Grains Genebank |  |
| 99 | **Yitpi** | 1999 | Australia | bread | C-8-MMC-8-HMM/FRAME | Australian Grains Genebank |  |
| 100 | Young | 2005 | Australia | bread | [VPM-1/3*BEULAH//SILVERSTAR](http://www.wheatpedigree.net/refer/ajaxShow/3810) | LongReach Plant Breeders |  |
|  |  |  |  |  |  |  |  |
| 1 | **Caparoi** | 2008 | Australia | durum | LY-2-6-3/930054 | Australian Grains Genebank |  |
| 2 | **DBA-Aurora** | 2014 | Australia | durum | is from the combination of two fixed lines. The first (maternal parent) of these is Tamaroi*2/Kalka//RH920318/ Kalka, which was crossed to the paternal parent Kalka*2/Tamaroi | Australian Grains Genebank |  |
| 3 | **Hyperno** | 2009 | Australia | durum | KALKA(SIB)/TAMAROI | Australian Grains Genebank |  |
| 4 | Kalka | 2004 | Australia | durum | LINGZHI-BAIMONG-BAIDAMAI/2*YALLAROI//RH-880009/3/WOLLAROI | University of Adelaide |  |
| 5 | Sahel-77 | 1977 | Algeria | durum | COCORIT-71(SIB)/4/TACUR-TIPO-125-E/4*TEHUACAN-67//2*ZENATI-BOUTEILLE/WELLS/3/(SIB)ANHINGA/5/RUFF(SIB)/(SIB)ALBE | Australian Grains Genebank |  |
| 6 | **Saintly** | 2009 | Australia | durum | KALKA(SIB,WLYY-9)*2/TAMAROI | Australian Grains Genebank |  |
| 7 | Tamaroi | 1998 | Australia | durum | ALTAR-84/4/TAM-1-B-17/KAMILAROI/3/WELLS/56111//GUILLEMOT | Commonwealth Scientific and Industrial Research Organisation |  |
| 8 | Tamaroi-*Nax2* | breeding line | Australia | durum | Tamaroi with sodium exclusion gene *Nax2* | Commonwealth Scientific and Industrial Research Organisation |  |
| 9 | **Tjilkuri** | 2010 | Australia | durum | BRND-Y-DURAY-2/R-875-LYT//LY-TM; BRINDUR/3/YALLAROI*2//DUR-A/YALLAROI/4/RAC-875/KALKA//TAMAROI/5/LINGHZI/YALLAROI//TAMAROI/3/LINGHZI/YALLAROI | Australian Grains Genebank |  |
| 10 | WID 902 | breeding line | Australia | durum | from a random cross made in the birdcage designated CIL/23*23/11 ; bic(ZbLYTTm#/7)* WNa49LY)/1 | University of Adelaide |  |
| 11 | **WID802** | 2012 | Australia | durum | SYR-YTLYD/R-875-LYT//LY-TM; SYRICA-1/YALLAROI//TAMAROI/LINGZHI/2*YALLAROI/4/RAC-875/KALKA//TAMAROI/5/LINGHZI/YALLAROI//TAMAROI/3/LINGHZI/YALLAROI | Australian Grains Genebank |  |
| 12 | **Yawa** | 2012 | Australia | durum | [WT-LYLYT/R-875-LYT//LY-TM; WESTONIA/KALKA//KALKA/TAMAROI/3/RAC-875/KALKA//TAMAROI](http://www.wheatpedigree.net/refer/ajaxShow/4172) | Australian Grains Genebank |  |
|  |  |  |  |  |  |  |  |
| Commercial cultivars grown in South Australia in 2015 are given in bold font (https://grdc.com.au/__data/assets/pdf_file/0020/109055/sa-sowing-guide-2015-pdf.pdf.pdf). | | | | | |  |  |

| **Table S2** Accession number/probe set, annotation/gene function**,** primer sequence, amplicon of candidate genes, previously published genes and housekeeping genes investigated in Experiment 4. | | | | |
| --- | --- | --- | --- | --- |
| **Accession number/probe set** | **Annotation/predicted function** | **Primer sequence (5'-3')** | **Amplicon (bp)** | **Reference** |
| *Candidate genes identified in the present study* | |  |  |  |
| TraesCS4D01G343200.1 | Calcium-transporting ATPase_chr4D | Fwd GTACCGAGGTTTTGCTAATTC | 121 | GWAS in Experiment 1 |
|  |  | Rev CGGAGCTGCATGTTGTGAAG |  |  |
| TraesCS4D01G344200.1 | Na(+)/H(+) antiporter NhaB_chr4D | Fwd GGTTTTAGGTTTGATTAGTC | 119 | GWAS in Experiment 1 |
|  |  | Rev CCATTTGCATAATGTATACAC |  |  |
|  |  |  |  |  |
|  |  |  |  |  |
| TraesCS4D01G344300.1 | Aquaporin_chr4D (Aquaporin-like protein TIF1-4) | Fwd CGCCGGGTTCGTCTATGAAAC | 98 | GWAS in Experiment 1 |
|  |  | Rev GCAAGTATGCGAGCAACCTG |  |  |
|  |  |  |  |  |
|  |  |  |  |  |
| *Candidate genes reported previously* | |  |  |  |
| XM020307045/Ta.22954.1.S1 | mRNA | Fwd CCCAGCAAGTACAAGCATCC | 62 | Mott and Wang (2007) |
|  |  | Rev CGTAAAATAGGTATCGACCG |  |  |
| AK4553054/TaAffx.56132.1.S1 | Putative high-affinity potassium transporter | Fwd CGTATACGCCTTCCTCAGG | 71 | Mott and Wang (2007) |
|  |  | Rev GCAGCTGGCTTCTTGGGATG |  |  |
| AK4544458/TaAffx.25629.1.S1 | Vacuolar pyrophosphatase similar to AVP1 (*TaAVP1*) | Fwd GACCGGTCTTGCCATTGATG | 162 | Mott and Wang (2007) |
|  |  | Rev CTGAGCCAATTGCGAATCCC |  |  |
| *Published genes* |  |  |  |  |
| EF062819 | Putative sodium transporter (*TmHKT1_4_A2*) | Fwd GGAGACACAGGGTATCAGGC | 102 | Tounsi *et al*. (2016) |
|  |  | Rev AGCTTCTCCCTCTCGGTGAC |  |  |
| AY296910 | Na+/H+ antiporter (*NHX1*) | Fwd GCCTGGTTCACCCATAGAGA | 159 | Mott and Wang (2007 |
|  |  | Rev CACCGAAAGAATCCCAAGAG |  |  |
| AY040246 | Na+/H+ antiporter (*NHX*2) | Fwd ATTTTGCTCGGGTTGGTTCTGGTT | 354 | Mott and Wang (2007) |
|  |  | Rev GTGCAGGGACTTCGGTGACGC |  |  |
| *Housekeeping genes* |  |  |  |  |
| KC775780 | Actin gene (TaActin) | Fwd GACAATGGAACCGGAATGGTC | 347 |  |
|  |  | Rev GTGTGATGCCAGATTTTCTCCAT |  |  |
| AK456344 | Glycilytic glyceraldehyde-3-phosphate dehydrogenase gene (TaGAPdH) | Fwd TTCAACATCATTCCAAGCAGCA | 220 |  |
|  |  | Rev CGTAACCCAAAATGCCCTTG |  |  |
| AK456000 | Eukaryotic elongation factor (TaEFA) | Fwd CAGATTGGCAACGGCTACG | 227 |  |
|  |  | Rev CGGACAGCAAAACGACCAAG |  |  |

**Table S3** Best linear unbiased estimates (BLUEs) for element concentrations (mg kg^-1^ DW) at heading in 100 bread wheat entries (*Triticum aestivum* L*.)* and 12 durum wheat entries *Triticum turgidum* subsp *durum)* grown under sodicity (8 g kg^-1^ Na^+^-humate) in Experiment 1 (n=4). Na^+^ concentration data were transformed to natural logarithms for analysis.

| Bread wheat | logNa^+^ | Na^+^ back-transformed | Cl^-^ | K^+^ | Ca^2+^ | Mg^2+^ |
| --- | --- | --- | --- | --- | --- | --- |
| A25 | 6.184 | 485 | 10759 | 37268 | 2852 | 1581 |
| AGT Katana | 7.001 | 1097 | 8270 | 35373 | 4387 | 1826 |
| AGT Scythe | 6.738 | 844 | 7357 | 36666 | 1832 | 1536 |
| Annuello | 6.624 | 753 | 10660 | 41789 | 2982 | 1371 |
| Anza | 6.438 | 625 | 11258 | 43972 | 2779 | 1371 |
| Aroona | 6.693 | 807 | 11419 | 42209 | 1645 | 1449 |
| Axe | 6.359 | 578 | 9387 | 40519 | 2422 | 1176 |
| AZDH1 | 7.006 | 1104 | 10306 | 38544 | 3650 | 1566 |
| Baart-46 | 7.792 | 2421 | 11822 | 33828 | 3005 | 1665 |
| Barham | 7.219 | 1366 | 12451 | 39556 | 2753 | 1570 |
| Beckom | 6.808 | 905 | 14278 | 40919 | 3034 | 1952 |
| Berkut | 6.492 | 660 | 6743 | 30698 | 2854 | 2146 |
| Bolac | 6.97 | 1064 | 9561 | 36787 | 2181 | 1174 |
| Brookton | 6.204 | 495 | 5370 | 36437 | 1861 | 1394 |
| Calingiri | 7.015 | 1114 | 9098 | 38516 | 2077 | 1677 |
| Carnamah | 6.036 | 418 | 10266 | 41166 | 3289 | 2311 |
| Cascades | 6.679 | 796 | 10849 | 40032 | 1945 | 1596 |
| Condor | 6.549 | 699 | 10700 | 38599 | 2427 | 1503 |
| Corack | 6.629 | 757 | 12196 | 40940 | 2742 | 1717 |
| Correll | 7.264 | 1429 | 11687 | 37811 | 2360 | 1437 |
| Cosmick | 7.274 | 1442 | 10527 | 36271 | 1763 | 1189 |
| Cutlass | 6.922 | 1014 | 10423 | 39773 | 2320 | 1579 |
| Drysdale | 6.911 | 1003 | 6468 | 33855 | 2617 | 1439 |
| DS Darwin | 6.459 | 638 | 9765 | 40511 | 2972 | 1876 |
| Ega-Eagle-Rock | 6.538 | 691 | 9124 | 41081 | 2590 | 1632 |
| Egret | 6.95 | 1043 | 7025 | 31400 | 1417 | 1618 |
| Emu Rock | 6.861 | 954 | 7479 | 38656 | 3438 | 1606 |
| Espada | 6.852 | 946 | 9856 | 46103 | 2952 | 1485 |
| Estoc | 6.769 | 870 | 7428 | 40131 | 2288 | 1635 |
| Excalibur | 6.664 | 784 | 7715 | 41885 | 2007 | 1750 |
| Fang | 6.336 | 564 | 10676 | 40801 | 2101 | 1651 |
| Federation | 7.853 | 2575 | 7821 | 31824 | 2112 | 1595 |
| Fortune | 6.782 | 882 | 10083 | 35702 | 2744 | 2067 |
| Frame | 7.302 | 1484 | 6501 | 35404 | 2330 | 1442 |
| Gabo | 6.592 | 729 | 9101 | 37731 | 1121 | 1273 |
| Gladius | 7.223 | 1370 | 11541 | 42364 | 2666 | 1497 |
| Grenade CL Plus | 6.57 | 713 | 7377 | 43299 | 2193 | 1417 |
| Halberd | 7.153 | 1278 | 10333 | 40524 | 1935 | 1280 |
| Harper | 6.665 | 784 | 6355 | 38274 | 2335 | 1527 |
| Hartog | 7.535 | 1873 | 10244 | 37420 | 3456 | 1717 |
| Hatchet CL Plus | 6.549 | 699 | 7737 | 39361 | 3043 | 1567 |
| Inia 66R | 6.761 | 864 | 8572 | 38711 | 2845 | 1624 |
| Janz | 7.043 | 1145 | 14427 | 45148 | 3154 | 2006 |
| Justica CL Plus | 6.217 | 501 | 9365 | 42800 | 3210 | 1719 |
| Kharchia-65 | 7.329 | 1525 | 11689 | 35821 | 3968 | 2287 |
| Kite | 6.57 | 714 | 12473 | 44615 | 2135 | 1509 |
| Kloka | 7.727 | 2269 | 9722 | 38063 | 3223 | 1305 |
| Kord CL Plus | 7.294 | 1471 | 12142 | 42456 | 2267 | 1474 |
| Krichauff | 5.518 | 249 | 8372 | 41264 | 2734 | 1562 |
| KRL-19 | 6.091 | 442 | 11480 | 39901 | 3495 | 1902 |
| Kukri | 6.873 | 966 | 7343 | 39260 | 1968 | 1335 |
| Lerma Rojo | 6.533 | 687 | 7042 | 33211 | 3430 | 1837 |
| Longreach Catalina | 6.239 | 512 | 12666 | 40561 | 4024 | 2022 |
| Longreach Cobra | 5.728 | 307 | 9251 | 40132 | 2616 | 1960 |
| Longreach Dart | 5.58 | 265 | 10898 | 38599 | 3037 | 1389 |
| Longreach Impala | 6.561 | 707 | 9882 | 38611 | 2813 | 1512 |
| Longreach Orion | 6.697 | 810 | 10811 | 43159 | 1885 | 1604 |
| Longreach Phantom | 6.855 | 949 | 9458 | 37150 | 2505 | 1782 |
| Longreach Scout | 7.205 | 1346 | 8175 | 38462 | 2387 | 1371 |
| Longreach Trojan | 6.621 | 751 | 8374 | 38595 | 2008 | 1650 |
| Mace | 6.595 | 731 | 9531 | 45139 | 2405 | 1729 |
| Machete | 6.255 | 521 | 7969 | 41509 | 853 | 1195 |
| Magenta | 7.202 | 1342 | 7797 | 37242 | 2015 | 1956 |
| Minto | 6.87 | 963 | 9613 | 45015 | 2238 | 1395 |
| Molineux | 7.012 | 1109 | 8038 | 36744 | 1734 | 1863 |
| MW#28 | 7.172 | 1303 | 7606 | 37443 | 2054 | 1585 |
| MW#293 | 9.874 | 19413 | 5422 | 26910 | 1383 | 1176 |
| MW#451 | 9.717 | 16600 | 5974 | 24820 | 1431 | 1449 |
| MW#491 | 6.829 | 924 | 9323 | 46270 | 2761 | 1644 |
| Nainari 60 | 7.133 | 1252 | 8672 | 36161 | 1068 | 939 |
| Olympic | 7.938 | 2802 | 5344 | 33298 | 1423 | 1275 |
| Peake | 5.582 | 266 | 12668 | 46103 | 3185 | 1360 |
| Pitic-62 | 7.649 | 2099 | 13250 | 36209 | 2690 | 1883 |
| RAC875 | 7.001 | 1097 | 13243 | 47295 | 2274 | 1114 |
| Sakha 61 | 7.067 | 1173 | 8033 | 38868 | 4932 | 1690 |
| Sakha 8 | 6.488 | 657 | 6637 | 38552 | 3806 | 2071 |
| Samnyt 16 | 7.339 | 1539 | 9658 | 36241 | 3471 | 1870 |
| Shasta | 5.974 | 393 | 8627 | 40844 | 2969 | 1742 |
| Shield | 6.783 | 883 | 8969 | 42242 | 2267 | 1699 |
| Sokoll | 7.128 | 1246 | 8414 | 35913 | 2869 | 1688 |
| Sonora-64 | 5.878 | 357 | 6452 | 36804 | 5237 | 2464 |
| Specter | 5.948 | 383 | 8105 | 37796 | 2648 | 2209 |
| Stylet | 6.712 | 822 | 5937 | 38289 | 2476 | 1758 |
| Suneca | 6.344 | 569 | 8687 | 34533 | 2694 | 1566 |
| Sunlin | 7.275 | 1443 | 10063 | 40497 | 1592 | 1261 |
| Tammarin-Rock | 6.864 | 957 | 10072 | 39035 | 2096 | 1274 |
| Trident | 6.452 | 634 | 6186 | 39097 | 2326 | 1488 |
| VM506 | 7.869 | 2616 | 7939 | 44026 | 3330 | 1561 |
| Warigal | 7.025 | 1124 | 8466 | 39228 | 1907 | 1655 |
| Warimek | 7.104 | 1217 | 8022 | 40447 | 2429 | 1578 |
| Wariquam | 7.173 | 1304 | 9686 | 37580 | 2821 | 1561 |
| Wentworth | 6.8 | 898 | 14506 | 48404 | 2924 | 1675 |
| Westonia | 4.795 | 121 | 6877 | 36757 | 3376 | 1514 |
| Westonia-*Nax1* | 4.704 | 110 | 8052 | 38885 | 3872 | 1556 |
| Westonia-*Nax2* | 3.829 | 46 | 7769 | 37619 | 3098 | 1447 |
| Worrakatta | 7.018 | 1116 | 10044 | 42300 | 3027 | 1231 |
| Wyalkatchem | 6.097 | 444 | 11528 | 43100 | 3007 | 1536 |
| Yecora Rojo | 6.942 | 1035 | 10831 | 42345 | 3703 | 1461 |
| Yitpi | 7.217 | 1362 | 5327 | 34433 | 2074 | 1142 |
| Young | 6.51 | 672 | 10128 | 41209 | 3026 | 1234 |
| Durum wheat |  |  |  |  |  |  |
| Caparoi | 10.008 | 22194 | 7170 | 19197 | 1505 | 534 |
| DBA-Aurora | 9.925 | 20440 | 6900 | 23355 | 1281 | 524 |
| Hyperno | 9.82 | 18390 | 7443 | 25222 | 1094 | 512 |
| Kalka | 9.951 | 20968 | 6962 | 22565 | 1290 | 523 |
| Sahel-77 | 10.111 | 24624 | 9162 | 16883 | 1112 | 528 |
| Saintly | 10.071 | 23650 | 8061 | 12750 | 1383 | 423 |
| Tamaroi | 10.272 | 28909 | 9573 | 12536 | 1162 | 358 |
| Tamaroi-*Nax2* | 8.285 | 3965 | 7543 | 39237 | 3044 | 768 |
| Tjilkuri | 10.252 | 28345 | 8378 | 13555 | 1057 | 384 |
| WID 802 | 10.108 | 24535 | 10832 | 18129 | 1052 | 358 |
| WID 902 | 6.436 | 624 | 7680 | 38201 | 3200 | 773 |
| Yawa | 9.653 | 15565 | 6946 | 26066 | 1240 | 540 |
|  |  |  |  |  |  |  |
| **HSD** | **0.847** |  | **3502** | **6694** | **1143** | **367** |
|  |  |  |  |  |  |  |
|  | logNa | Na back-transformed | Cl^-^ | K^+^ | Ca^2+^ | Mg^2+^ |
| Bread wheat |  |  |  |  |  |  |
| Mean | 6.771 | 1308 | 9296 | 39057 | 2627 | 1592 |
| Range | 3.829-9.874 | 46-19413 | 5327-14506 | 24820-48404 | 853-5237 | 939-2464 |
|  |  |  |  |  |  |  |
| Durum wheat |  |  |  |  |  |  |
| Mean | 9.574 | 19351 | 8054 | 22308 | 1535 | 519 |
| Range | 6.436-10.272 | 624-28909 | 6900-10832 | 12536-39237 | 1052-3200 | 358-773 |

**Table S4** SNPs significantly associated with leaf Na^+^ concentration and their flanking candidate genes identified in Experiment 1. Candidate genes selected for expression analyses are presented in bold.

| 90K marker name | Chromosome | Position^a^ | *P*-value | Gene located within 1Mb left and right | Gene annotation with potential function on Na^+^ accumulation | Pfam-ID |
| --- | --- | --- | --- | --- | --- | --- |
| RAC875_c67855_529 | 4D | 499,108,852 | 4.02E-05 | From TraesCS4D01G341500.1 to TraesCS4D01G345000.1 | **Calcium-transporting ATPase^b^**  **Na^(+)^/H^(+)^ antiporter NhaB^b^**  **Aquaporin TIF1-4^b^** | PF00689  PF06450  PF00230 |
| Kukri_c17964_383 | N.A. | N.A. | 5.13E-05 | N.A. | N.A. | N.A. |
| Kukri_c86597_51 | N.A. | N.A. | 5.58E-05 | N.A. | N.A. | N.A. |
| RAC875_c43124_345 | 7A | 12,910,700 | 5.97E-05 | From TraesCS7A01G029200.1 to TraesCS7A01G032300.1 | N.A. | N.A. |
| wsnp_RFL_Contig3911_4319047 | 2B | 793,584,220 | 6.11E-05 | From TraesCS2B01G613200.1 to TraesCS2B01G618700.2 | N.A. | N.A. |
| tplb0058o04_1071 | 2A | 762,646,301 | 6.18E-05 | From TraesCS2A01G558300.1 to TraesCS2A01G562500.1 | N.A. | N.A. |
| RAC875_c51375_299 | 4B | 652,373,327 | 7.17E-05 | From TraesCS4B01G362300.1 to TraesCS4B01G365200.1 | Aquaporin PIP2 | PF00230 |
| RAC875_c65269_290 | 5B | 702,606,243 | 7.41E-05 | From TraesCS5B01G550500.1 to TraesCS5B01G553600.1 | N.A. | N.A. |
| tplb0049b24_1152 | 2D | 643,436,190 | 7.44E-05 | From TraesCS2D01G580600.1 to TraesCS2D01G585400.1 | N.A. | N.A. |

^a^ SNP positions according to IWGSC RefSeq v1.0.

**Table S5** Allelic effects on leaf Na^+^ concentration of SNP markers. The alleles for two high-Na^+^ entries, MW#293 and MW#451, were the same. Log-transformed and back-transformed values are presented.

|  |  | Log-Na | Log-Na | Back-transformed-Na | Back-transformed-Na | MW#451/  MW#293 allele |
| --- | --- | --- | --- | --- | --- | --- |
| SNP | Ch.* | AA | BB | AA | BB |  |
| RAC875_c67855_529 | 4D | 6.6 | 8.9 | 880 | 12420 | BB |
| Kukri_c17964_383 | n/a | 6.6 | 8.9 | 859 | 12420 | BB |
| Kukri_c86597_51 | n/a | 6.6 | 8.9 | 907 | 12420 | BB |
| RAC875_c43124_345 | 7A | 8.1 | 6.1 | 7019 | 864 | AA |
| wsnp_RFL_Contig3911_4319047 | 2B | 9.8 | 6.9 | 18006 | 1109 | AA |
| tplb0058o04_1071 | 2A | 6.4 | 6.8 | 821 | 957 | n/a |
| RAC875_c51375_299 | 4B | 8.2 | 6.7 | 7097 | 927 | AA |
| RAC875_c65269_290 | 5B | 6.9 | 9.8 | 1067 | 18006 | BB |
| tplb0049b24_1152 | 2D | 6.8 | 6.5 | 967 | 863 | n/a |

n/a; not available

**Table S6** Slopes of incremental water use over time (surrogate for growth rate) derived from linear regressions up to heading stage in bread wheat (*Triticum aestivum* L*.)* cv. Westonia, Westonia-*Nax1*, Westonia-*Nax2* and Baart-46 under salinity and sodicity in Experiment 2 (n=4).

|  |  | | | |
| --- | --- | --- | --- | --- |
| Salinity  (mM NaCl) | Westonia | West-*Nax1* | West-*Nax2* | Baart-46 |
| 0 | 3.8 | 4.3 | 3.8 | 5.4 |
| 50 | 2.6 | 2.8 | 2.7 | 4.3 |
| 100 | 2.0 | 1.9 | 1.8 | 2.7 |
| 150 | 1.4 | 1.5 | 1.3 | 1.8 |
| 200 | 0.8 | 1.0 | 0.8 | 1.2 |
| LSD* |  | 0.5 | |  |
|  |  |  | |  |
| Sodicity (g kg^-1^ soil Na^+^-humate) | Westonia | West-*Nax1* | West-*Nax2* | Baart-46 |
| 0 | 3.7 | 4.1 | 3.7 | 5.4 |
| 2.0 | 3.6 | 3.7 | 3.4 | 5.4 |
| 4.0 | 3.3 | 3.5 | 3.1 | 5.3 |
| 8.0 | 2.6 | 2.8 | 2.2 | 4.0 |
| 16.0 | 1.5 | 1.6 | 1.4 | 2.4 |
| LSD* |  | 0.5 | |  |

*LSD refers to Least Significant Difference at *P*=0.05 for species x treatment interaction

**Table S7** Best linear unbiased estimates for leaf Na^+^, K^+^, Ca^2+^, Mg^2+^ and Cl^-^ concentrations (mg kg^-1^ DW) in bread wheat (*Triticum aestivum* L*.)* cv. Westonia, Westonia-*Nax1*, Westonia-*Nax2* and Baart-46 under salinity and sodicity in Experiment 2 (n=4).

|  |  | LogNa^+^* |  |  |  |  | | K^+^ |  | |  | |  | |  | | Ca^2+^ | |  |  |  |  | Mg^2+^ |  |  |  |  | LogCl^-^* |  |  |
| --- | --- | --- | --- | --- | --- | --- | --- | --- | --- | --- | --- | --- | --- | --- | --- | --- | --- | --- | --- | --- | --- | --- | --- | --- | --- | --- | --- | --- | --- | --- |
| Salinity  (mM NaCl) | Westonia | West-*Nax1* | West-*Nax2* | Baart-46 |  | Westonia | | West-*Nax1* | West-*Nax2* | | Baart-46 | |  | | Westonia | | West-*Nax1* | | West-*Nax2* | Baart-46 |  | Westonia | West-*Nax1* | West-*Nax2* | Baart-46 |  | Westonia | West-  *Nax1* | West-  *Nax2* | Baart-46 |
| 0 | 2.722 | 2.525 | 2.656 | 5.430 |  | 40287 | 39061 | | 39879 | 34218 | |  | | 5756 | | 6540 | | 7704 | | 6226 |  | 1952 | 2307 | 2182 | 2462 |  | 8.260 | 8.310 | 8.269 | 8.397 |
| 50 | 2.861 | 2.967 | 2.860 | 6.304 |  | 39132 | 36755 | | 40141 | 34746 | |  | | 7691 | | 7090 | | 8176 | | 7059 |  | 1723 | 1738 | 1769 | 2100 |  | 9.556 | 9.500 | 9.646 | 9.870 |
| 100 | 3.293 | 2.928 | 2.957 | 6.466 |  | 40792 | 37667 | | 40929 | 33610 | |  | | 7619 | | 6807 | | 7932 | | 7139 |  | 1674 | 1596 | 1618 | 1790 |  | 9.913 | 9.800 | 9.931 | 10.138 |
| 150 | 3.468 | 3.152 | 3.112 | 6.584 |  | 40565 | 42681 | | 40238 | 36637 | |  | | 7378 | | 7263 | | 7769 | | 7708 |  | 1427 | 1490 | 1558 | 1554 |  | 10.051 | 10.081 | 9.990 | 10.346 |
| 200 | 3.444 | 3.293 | 3.272 | 6.539 |  | 38349 | 40453 | | 39440 | 36447 | |  | | 7128 | | 7645 | | 7318 | | 6915 |  | 1329 | 1408 | 1386 | 1349 |  | 10.077 | 10.065 | 10.018 | 10.385 |
| LSD** |  | 0.305 |  |  |  |  | | 2320 |  | |  | |  | |  | | 862 | |  |  |  |  | 135 |  |  |  |  | 0.110 |  |  |
|  |  |  |  |  |  |  | |  |  | |  | |  | |  | |  | |  |  |  |  |  |  |  |  |  |  |  |  |
|  |  | LogNa^+^* |  |  |  |  | | K^+^ |  | |  | |  | |  | | Ca^2+^ | |  |  |  |  | Mg^2+^ |  |  |  |  | LogCl^-^* |  |  |
| Sodicity  (g kg^-1^ soil Na^+^-humate) | Westonia | West-*Nax1* | West-*Nax2* | Baart-46 |  | Westonia | | West-*Nax1* | West-*Nax2* | | Baart-46 | |  | | Westonia | | West-*Nax1* | | West-*Nax2* | Baart-46 |  | Westonia | West-*Nax1* | West-*Nax2* | Baart-46 |  | Westonia | West-  *Nax1* | West-*Nax2* | Baart-46 |
| 0 | 2.722 | 2.525 | 2.656 | 5.430 |  | 40287 | | 39061 | 39879 | | 34218 | |  | | 5756 | | 6540 | | 7704 | 6226 |  | 1952 | 2307 | 2182 | 2462 |  | 8.260 | 8.310 | 8.269 | 8.397 |
| 2 | 2.738 | 2.685 | 2.690 | 6.669 |  | 40861 | | 41077 | 43036 | | 36832 | |  | | 5041 | | 5115 | | 6172 | 4825 |  | 1730 | 2032 | 1924 | 2333 |  | 8.524 | 8.516 | 8.576 | 8.651 |
| 4 | 3.896 | 2.838 | 2.846 | 7.107 |  | 42188 | | 42140 | 43703 | | 38079 | |  | | 4120 | | 4163 | | 4958 | 3543 |  | 1738 | 1997 | 1780 | 1982 |  | 8.676 | 8.741 | 8.822 | 8.877 |
| 8 | 4.921 | 3.691 | 3.214 | 7.227 |  | 43877 | | 42081 | 43708 | | 37564 | |  | | 3624 | | 3598 | | 4160 | 3016 |  | 1683 | 1694 | 1712 | 1708 |  | 8.997 | 8.999 | 8.975 | 9.305 |
| 16 | 6.301 | 5.186 | 5.479 | 7.777 |  | 41599 | | 41985 | 42496 | | 36909 | |  | | 2932 | | 3477 | | 2894 | 2774 |  | 1502 | 1690 | 1507 | 1524 |  | 8.969 | 9.141 | 9.034 | 9.583 |
| LSD** |  | 0.338 |  |  |  |  | | 2526 |  | |  | |  | |  | | 654 | |  |  |  |  | 178 |  |  |  |  | 0.118 |  |  |
|  |  |  |  |  |  |  | |  |  | |  | |  | |  | |  | |  |  |  |  |  |  |  |  |  |  |  |  |

*Na^+^ and Cl^-^ concentration data were transformed to natural logarithms. **LSD refers to Least Significant Difference at *P*=0.05 for species x treatment interaction. Although control treatment appeared once for both treatments in the experimental design, the control values are presented for both treatments separately to assist comparisons within each treatment. However, in statistical analysis, all data were analysed together with the control treatment appearing once.

**Table S8** Back-transformed Na^+^ and Cl^-^ concentrations in bread wheat (*Triticum aestivum* L*.)* cv. Westonia, Westonia-*Nax1*, Westonia-*Nax2* and Baart-46 under different levels of salinity and sodicity in Experiment 2 (n=4).

|  | Na^+^ concentration (mg kg^-1^ DW) | | | |  | Cl^-^ concentration (mg kg^-1^ DW) | | | |
| --- | --- | --- | --- | --- | --- | --- | --- | --- | --- |
| Treatment |  |  |  |  |  |  |  |  |  |
| Salinity  (mM NaCl) | Westonia | West-*Nax1* | West-*Nax2* | Baart-46 |  | Westonia | West-*Nax1* | West-*Nax2* | Baart-46 |
| 0 | 15 | 12 | 14 | 228 |  | 3865 | 4066 | 3900 | 4433 |
| 50 | 17 | 19 | 17 | 547 |  | 14134 | 13355 | 15452 | 19350 |
| 100 | 27 | 19 | 19 | 643 |  | 20192 | 18028 | 20562 | 25287 |
| 150 | 32 | 23 | 22 | 724 |  | 23175 | 23885 | 21816 | 31132 |
| 200 | 31 | 27 | 26 | 692 |  | 23779 | 23506 | 22422 | 32356 |
|  |  |  |  |  |  |  |  |  |  |
|  |  |  |  |  |  |  |  |  |  |
| Sodicity  (g kg^-1^ soil Na^+-^humate) | Westonia | West-*Nax1* | West-*Nax2* | Baart-46 |  | Westonia | West-*Nax1* | West-*Nax2* | Baart-46 |
| 0 | 15 | 12 | 14 | 228 |  | 3865 | 4066 | 3900 | 4433 |
| 2 | 15 | 15 | 15 | 787 |  | 5034 | 4996 | 5305 | 5713 |
| 4 | 49 | 17 | 17 | 1221 |  | 5859 | 6253 | 6785 | 7168 |
| 8 | 137 | 40 | 25 | 1376 |  | 8079 | 8093 | 7901 | 10990 |
| 16 | 545 | 179 | 240 | 2384 |  | 7854 | 9327 | 8384 | 14513 |
|  |  |  |  |  |  |  |  |  |  |

Although control treatment appeared once for both treatments in the experimental design, the control values were presented for both treatments separately to assist comparisons within each treatment. However, in statistical analysis, all data were analysed together with the control treatment appearing once.

**Table S9** Slopes of incremental water use over time (surrogate for growth rate) derived from linear regressions up to heading stage in 20 bread wheat entries (*Triticum aestivum* L.), three durum wheat entries (*Triticum turgidum* subsp *durum* cv. Tamaroi , Tamaroi-*Nax2,* and Yawa) and one barley cultivar (*Hordeum vulgare* L. cv. Clipper) under control, salinity (100 mM NaCl) and sodicity (8 g kg^-1^ Na^+^-humate) in Experiment 3 (n=4).

|  | Control |  | Sodicity |  | Salinity |
| --- | --- | --- | --- | --- | --- |
| AGT Katana | 2.8 |  | 1.1 |  | 1.1 |
| Axe | 2.1 |  | 0.9 |  | 0.7 |
| Krichauff | 2.7 |  | 1.0 |  | 1.2 |
| Longreach Cobra | 3.0 |  | 1.0 |  | 1.3 |
| Baart-46 | 4.5 |  | 2.2 |  | 2.4 |
| Beckom | 3.2 |  | 1.8 |  | 1.4 |
| Mace | 3.3 |  | 1.5 |  | 1.4 |
| MW#293 | 4.7 |  | 3.9 |  | 3.7 |
| Correll | 3.2 |  | 1.3 |  | 1.4 |
| Condor | 4.5 |  | 2.6 |  | 2.4 |
| MW#451 | 3.3 |  | 1.4 |  | 1.5 |
| Pitic-62 | 4.3 |  | 2.3 |  | 2.1 |
| Drysdale | 3.0 |  | 1.0 |  | 1.2 |
| Federation | 6.0 |  | 3.4 |  | 2.7 |
| Halberd | 4.6 |  | 1.8 |  | 1.8 |
| Hartog | 2.9 |  | 1.0 |  | 1.3 |
| Kharchia-65 | 4.9 |  | 3.0 |  | 2.4 |
| Westonia | 4.0 |  | 1.5 |  | 1.6 |
| Wyalkatchem | 2.4 |  | 1.1 |  | 1.1 |
| Yitpi | 3.7 |  | 1.5 |  | 1.6 |
|  |  |  |  |  |  |
| Tamaroi | 4.6 |  | 1.8 |  | 2.0 |
| Tamaroi-*Nax2* | 4.5 |  | 2.4 |  | 1.7 |
| Yawa | 5.1 |  | 3.0 |  | 2.9 |
|  |  |  |  |  |  |
| Clipper | 5.6 |  | 2.4 |  | 2.8 |
| LSD cont vs sodicity |  | 0.8 |  |  |  |
| LSD cont vs salinity |  |  |  | 0.8 |  |
|  |  |  |  |  |  |

*LSD refers to Least Significant Difference test value at *P*=0.05 for species x treatment interaction.

**Table S10** Best linear unbiased estimates for leaf K^+^, Ca^2+^ and Mg^2+^ concentrations (mg kg^-1^ DW) in 20 bread wheat entries (*Triticum aestivum* L.), three durum wheat entries (*Triticum turgidum* subsp *durum* cv. Tamaroi, Tamaroi-*Nax2,* and Yawa) and one barley cultivar (*Hordeum vulgare* L.cv. Clipper) under control, salinity (100 mM NaCl) and sodicity (8 g kg^-1^ Na^+^-humate) in Experiment 3 (n=4).

|  |  | | | K^+^ |  | |  |  | | Ca^2+^ |  |  | | | Mg^2+^ |  |
| --- | --- | --- | --- | --- | --- | --- | --- | --- | --- | --- | --- | --- | --- | --- | --- | --- |
|  |  | control | sodicity | | | salinity | |  | control | sodicity | salinity | |  | control | sodicity | salinity |
| Longreach Cobra |  | 36195 | 40803 | | | 35571 | |  | 7256 | 3348 | 8420 | |  | 2841 | 1897 | 2271 |
| Westonia |  | 35116 | 42279 | | | 33991 | |  | 8171 | 3785 | 7174 | |  | 2527 | 1622 | 1889 |
| Krichauff |  | 37910 | 43897 | | | 39750 | |  | 6638 | 2628 | 5842 | |  | 2458 | 1292 | 1618 |
| Mace |  | 41062 | 45004 | | | 41691 | |  | 5393 | 2319 | 5246 | |  | 2597 | 1498 | 1693 |
| Wyalkatchem |  | 36371 | 43430 | | | 39166 | |  | 8055 | 3192 | 8515 | |  | 2881 | 1607 | 1953 |
| Axe |  | 42832 | 45870 | | | 45646 | |  | 6302 | 2579 | 6241 | |  | 2156 | 1181 | 1503 |
| Halberd |  | 42081 | 41028 | | | 38207 | |  | 6809 | 2290 | 7873 | |  | 2479 | 1270 | 1994 |
| Beckom |  | 44062 | 48719 | | | 44317 | |  | 5976 | 2793 | 7558 | |  | 2775 | 1612 | 2179 |
| Yitpi |  | 34783 | 39531 | | | 33095 | |  | 5961 | 1755 | 7784 | |  | 2570 | 1313 | 2026 |
| Condor |  | 36922 | 42016 | | | 35453 | |  | 8415 | 3061 | 9240 | |  | 2987 | 1658 | 2376 |
| Kharchia-65 |  | 30194 | 34456 | | | 24958 | |  | 7983 | 4563 | 9971 | |  | 2851 | 1931 | 2939 |
| AGT Katana |  | 38951 | 43964 | | | 37232 | |  | 9212 | 4056 | 8290 | |  | 2744 | 1676 | 1967 |
| Drysdale |  | 35883 | 36594 | | | 35793 | |  | 5687 | 3017 | 6953 | |  | 2475 | 1605 | 1803 |
| Pitic-62 |  | 31681 | 36493 | | | 32557 | |  | 7995 | 3735 | 9150 | |  | 2949 | 1798 | 2539 |
| Correll |  | 38771 | 41952 | | | 37019 | |  | 6890 | 2468 | 7346 | |  | 2742 | 1387 | 1933 |
| Hartog |  | 35614 | 37094 | | | 34640 | |  | 9058 | 3827 | 8337 | |  | 2710 | 1881 | 2134 |
| Federation |  | 32973 | 34989 | | | 29931 | |  | 7853 | 2905 | 8472 | |  | 2930 | 1769 | 2459 |
| Baart-46 |  | 33777 | 37723 | | | 32390 | |  | 7643 | 3756 | 7014 | |  | 2509 | 1606 | 1911 |
| MW#293 |  | 36460 | 28490 | | | 31981 | |  | 4549 | 1343 | 5094 | |  | 2229 | 1135 | 1530 |
| MW#451 |  | 38135 | 27516 | | | 34032 | |  | 5615 | 1759 | 5588 | |  | 2421 | 1419 | 1831 |
|  |  |  |  | | |  | |  |  |  |  | |  |  |  |  |
| Tamaroi-*Nax2* |  | 39468 | 39393 | | | 42829 | |  | 8042 | 4102 | 7634 | |  | 1784 | 1188 | 1444 |
| Yawa |  | 37015 | 21706 | | | 32653 | |  | 7556 | 2383 | 8613 | |  | 1764 | 762 | 1542 |
| Tamaroi |  | 38756 | 16836 | | | 31415 | |  | 7780 | 2189 | 7512 | |  | 1803 | 665 | 1347 |
|  |  |  |  | | |  | |  |  |  |  | |  |  |  |  |
| Clipper |  | 30070 | 18646 | | | 19045 | |  | 10572 | 2960 | 10977 | |  | 1844 | 724 | 1477 |
| LSD control vs sodicity |  |  | 3092 | | |  | |  |  | 908 |  | |  |  | 200 |  |
| LSD control vs salinity |  |  |  | | | 2916 | |  |  |  | 1381 | |  |  |  | 232 |

LSD refers to Least Significant Difference at *P*=0.05 for species x treatment interaction.
